# Long-term expansion, genomic stability and in vivo safety of adult human pancreas organoids

**DOI:** 10.1186/s12861-020-0209-5

**Published:** 2020-02-26

**Authors:** Nikitas Georgakopoulos, Nicole Prior, Brigitte Angres, Gianmarco Mastrogiovanni, Alex Cagan, Daisy Harrison, Christopher J. Hindley, Robert Arnes-Benito, Siong-Seng Liau, Abbie Curd, Natasha Ivory, Benjamin D. Simons, Inigo Martincorena, Helmut Wurst, Kourosh Saeb-Parsy, Meritxell Huch

**Affiliations:** 1 The Wellcome Trust/ Cancer Research UK Gurdon Institute, University of Cambridge, Tennis Court Road, Cambridge, CB2 1QN UK; 2 Cambridge Biorepository for Translational Medicine & Department of Surgery, University o.f Cambridge, and NIHR Cambridge Biomedical Research Centre, Cambridge, CB2 0QQ UK; 3 Max Planck Institute of Molecular Cell Biology and Genetics, 01307 Dresden, Germany; 4 Cellendes GmbH, 72770 Reutlingen, Germany; 5 Wellcome Sanger Institute, Hinxton, Cambridgeshire, CB10 1SA UK; 6 Department of Physics, The Cavendish Laboratory, University of Cambridge, Thompson Avenue, Cambridge, JJ CB3 0HE UK; 7 Hepatopancreatobiliary Surgical Unit, Addenbrooke’s Hospital and MRC Cancer Unit, University of Cambridge, Cambridge, CB2 0XZ UK

**Keywords:** Pancreas, Organoid, In vivo safety, Chemically defined hydrogel, Genetic stability, Primary cultures

## Abstract

**Background:**

Pancreatic organoid systems have recently been described for the in vitro culture of pancreatic ductal cells from mouse and human. Mouse pancreatic organoids exhibit unlimited expansion potential, while previously reported human pancreas organoid (hPO) cultures do not expand efficiently long-term in a chemically defined, serum-free medium. We sought to generate a 3D culture system for long-term expansion of human pancreas ductal cells as hPOs to serve as the basis for studies of human pancreas ductal epithelium, exocrine pancreatic diseases and the development of a genomically stable replacement cell therapy for diabetes mellitus.

**Results:**

Our chemically defined, serum-free, human pancreas organoid culture medium supports the generation and expansion of hPOs with high efficiency from both fresh and cryopreserved primary tissue. hPOs can be expanded from a single cell, enabling their genetic manipulation and generation of clonal cultures. hPOs expanded for months in vitro maintain their ductal morphology, biomarker expression and chromosomal integrity. Xenografts of hPOs survive long-term in vivo when transplanted into the pancreas of immunodeficient mice. Notably, mouse orthotopic transplants show no signs of tumorigenicity. Crucially, our medium also supports the establishment and expansion of hPOs in a chemically defined, modifiable and scalable, biomimetic hydrogel.

**Conclusions:**

hPOs can be expanded long-term, from both fresh and cryopreserved human pancreas tissue in a chemically defined, serum-free medium with no detectable tumorigenicity. hPOs can be clonally expanded, genetically manipulated and are amenable to culture in a chemically defined hydrogel. hPOs therefore represent an abundant source of pancreas ductal cells that retain the characteristics of the tissue-of-origin, which opens up avenues for modelling diseases of the ductal epithelium and increasing understanding of human pancreas exocrine biology as well as for potentially producing insulin-secreting cells for the treatment of diabetes.

## Background

The pancreas exhibits dual functions: on the one hand, acinar and ductal cells act as an exocrine organ which aids in digestion, whilst on the other hand, pancreatic β cells, which are located in the islets of Langerhans together with α, δ, ε and PP cells, perform an endocrine function by regulating blood glucose levels through the secretion of insulin [1]. Both endocrine and exocrine cells are derived during development from the ventral and dorsal *PDX1+* foregut endoderm, which fuse to give rise to the head, body and tail of the pancreas. After pancreatic foregut specification, multipotent progenitors diverge into tip and trunk progenitors. Tip progenitors differentiate into acinar cells while bipotent trunk progenitors further give rise to ductal and endocrine cells [1, 2].

Exocrine pancreas disorders include pancreatic insufficiency, pancreatitis, pancreas cancer and cystic fibrosis. Diabetes mellitus is the most common disease of the endocrine pancreas, leading to aberrant regulation of blood glucose levels. In the case of Type 1 Diabetes (T1D), β cells are targeted and compromised by an autoimmune reaction. Solid pancreas and islet transplants are the gold standard curative treatments of T1D due to the restoration of a functional pool of cells. However, there is a shortage of suitable donor organs for transplantation. Expansion of islets in vitro would be an ideal treatment strategy; however, this remains a challenge due to the low proliferative capacity of mature endocrine cells and the tendency of islets to undergo epithelial to mesenchymal transition in culture [3]. Notably, ductal pancreas cells retain some degree of plasticity and can give rise, in some circumstances, to endocrine cells in vitro [4–7] and in vivo [8–10]. Therefore, human ductal cells could serve as a starting material for modelling pancreas ductal diseases ex vivo as well as for the derivation of glucose-responsive insulin-producing cells, provided they can be efficiently expanded in vitro.

In order for any given cellular source to serve for disease modelling as well as for a regenerative cell therapy, there are a number of criteria to fulfil; these include the generation of a large number of cells and demonstration of their genetic and transcriptomic stability over time. Additionally, in order for a cell therapy to translate into the clinic, production under Good Manufacturing Practice *(*GMP*)* conditions with a chemically defined medium, as well as safety of the product, must be demonstrated. Pluripotent stem cells (PSCs: either ESCs or iPSCs [11–14]) have attracted much attention as a source material both for pancreas disease modelling as well as for cell therapies to treat diabetes. However, the high mutation rates of PSCs in vitro and predisposition to form teratomas in vivo*,* upon transplantation, warrants concern over the use of these cells for therapies in the clinic [15, 16]. In contrast, epithelial organoids derived from adult tissues such as the liver [17], colon, stomach and prostate [18] exhibit a high degree of genomic integrity, with very low base substitution rates in coding regions. Indeed, Whole Genome Sequencing (WGS) of clonally expanded human liver organoid cultures demonstrated that 10-fold fewer mutations arose during long-term expansion of organoids compared with iPSC cultures [17]. Hence, efficient expansion of adult human pancreatic tissue has the potential to mitigate the limitations of ESC/iPSC-derived disease modelling and the safety and genetic stability hurdles for cell therapies, in part because the cells do not have to revert to a pluripotent-state.

The culture of human primary ductal cells is not trivial, and early studies failed to expand material past 1–2 weeks [4, 19]. Utilising 3D culture techniques, we established adult pancreas organoids from mouse pancreatic ducts that could be expanded long-term in vitro while also maintaining the capability to undergo endocrine differentiation in vivo [20]. Since then, we and others have adapted the culture system in order to generate adult human primary pancreas tissue ductal organoids [21–23]. Despite this success, efficient long-term expansion of adult human pancreas organoids (hPOs) and their clonal derivation has yet to be shown. In addition, long-term expansion from cryopreserved adult tissue, which would facilitate the cryo-banking of tissue material for subsequent cellular derivation, has not been achieved.

Here we report the long-term expansion of hPOs from both fresh and cryopreserved pancreas tissue from human donors, in a chemically defined, serum-free medium. We demonstrate their genomic stability in vitro, safety in vivo and their expansion potential in a chemically defined hydrogel. Our pancreas organoid model opens up the opportunity for establishing protocols for disease modelling for exocrine disorders as well as highlighting a potential cellular source for the future development of cell therapies for endocrine diseases such as T1D.

## Results

### Generation, long-term expansion and clonal derivation of human ductal pancreatic organoids

We and others have previously reported culture systems that support human ductal pancreatic organoid growth [21–23]. However, these suffer from several shortcomings in their application for disease modelling and cell therapy: (1) they do not support the long-term expansion required to generate the necessary cell numbers [22], (2) the medium compositions are not chemically defined and require the addition of serum to the medium [21, 23], 3) the extracellular matrix (ECM) used, namely Matrigel, suffers from batch-to-batch effects and additionally, is derived from mouse tumours, which makes it difficult to produce under GMP compliant conditions [21–23]. Hence, we first sought to develop a chemically defined medium that would support the long-term expansion of human primary ductal cells.

**Fig. 1:**
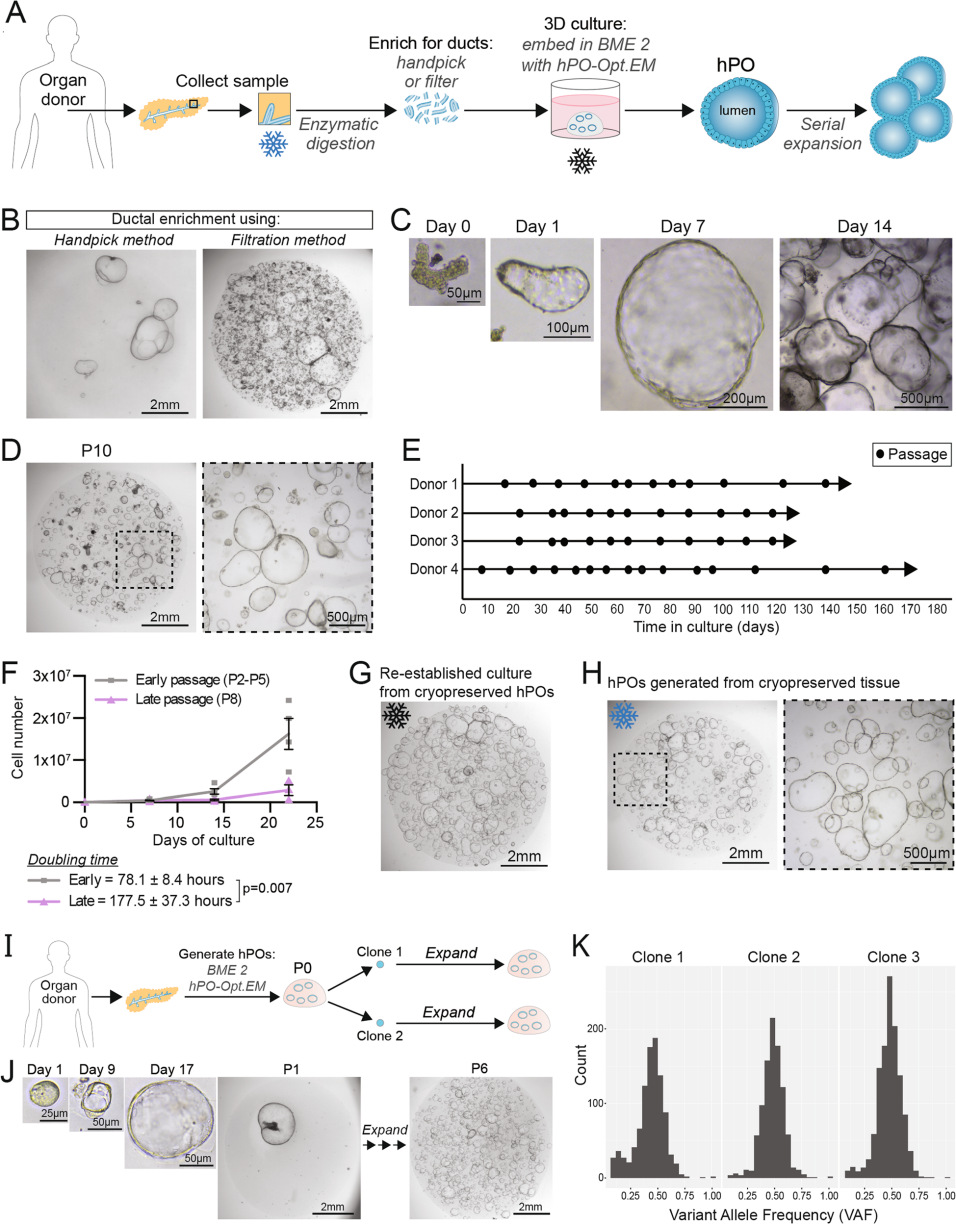
Human pancreatic organoids (hPOs) expand long-term, even from cryopreserved tissue and are amenable for clonal expansion. **a** Schematic of hPO generation and expansion. Pancreatic tissue undergoes enzymatic digestion to release ductal fragments, which are subsequently enriched either by handpicking or filtration. Pancreas ductal fragments are then embedded in BME 2 as extracellular matrix and overlayed with the hPO-Opt.EM medium (see methods; hPO-Opt.EM composition). Generated hPOs can be serially expanded by mechanical dissociation. Cryopreservation can be performed on the primary tissue for derivation at a later time (blue asterisk) or on the established hPOs (black asterisk). **b** Comparison of P0 cultures following ductal enrichment by handpicking (left) or filtration (right) (*n* = 5). **c** Brightfield images of ductal fragments isolated from fresh human pancreatic donor tissue grown and expanded as hPOs. **d** hPOs can be expanded and cultured long-term in vitro. Representative images of hPO culture at passage 10 (P10). **e** hPOs can be passaged over many months in hPO-Opt.EM. (*n* = 4 independent donors; circle = passage). **f** hPO growth curves indicate that hPOs expand exponentially even at late passages. Graph represents independent donors (early passage, grey *n* = 4; late passage, purple, *n* = 3), doubling time is indicated (78.1 ± 8.4 h at early passages). **g**) hPO cultures can be cryopreserved as hPO fragments as described in methods and re-stablished by embedding the fragments in BME 2 and overlayed with hPO-Opt.EM. hPOs derived from cryopreserved fragments generate new hPOs with same expansion rate as non-cryopreserved hPOs. Representative image of a P4 culture obtained from a hPO culture cryopreserved at P0 and kept in liquid N2 for 3 months (*n* = 9 independent donors). **h** hPOs can be generated from cryopreserved primary human pancreatic tissue (see methods for details). Image shows hPOs derived from a pancreas tissue that had been cryopreserved for 3 weeks (*n* = 3). **i** Workflow to generate clonal cultures from single hPO cells which are derived from P0 organoids. **j** Representative images showing the isolation of a single hPO cell to form a clonal organoid which can then be then clonally expanded long-term (*n* = 5 independent donors). **k** The variant allele frequency (VAF) of single nucleotide variants was assessed using genome sequencing data from three cultures derived from single cells as described in **i**), in all cases the VAF was close to 0.5, confirming clonality of these cultures

Human pancreas tissue samples were obtained from deceased transplant organ donors, enzymatically digested and isolated pancreatic ducts were seeded in Basement Membrane Extract Type 2 (BME 2) as ECM. A cocktail of growth factors and small molecule inhibitors were tested in different combinations and concentrations until we obtained an optimised culture medium that would support the expansion of human primary pancreas ductal cells beyond passage 10 (Fig. 1a). After several iterations to adapt our previously reported mouse culture conditions [20], we developed an optimised expansion medium for human pancreatic organoids (hPO-Opt.EM) by the combined addition of a TGFb inhibitor, Forskolin (FSK) and Prostglandin E2 (PGE2) together with an increased concentration of Rspo1. These factors were tested due to their use in the translation from mouse to human organoid cultures in other tissues, namely the liver (TGFb inhibitor and FSK) [17] and stomach (PGE2) [24]. This optimised serum-free culture medium supports the generation of hPOs with high efficiency (> 90%) (Additional file 2: Table S1) and facilitates their long-term expansion beyond 180 days in culture compared with previously published protocols (Additional file 1: Figure S1a). Isolation of ductal fragments can be conducted either by handpicking of ducts [23] or by filtration of the digested tissue. Handpicking of ducts results in a purer population of ductal organoid structures at P0; however, filtration is substantially faster (handpicking > 30 min vs. filtration ~ 5 min) and yields more organoids (Fig. 1b). Regardless of the ductal enrichment technique used, seeded ductal cells begin to proliferate and rapidly form cystic organoids by day 7, which are ready to passage by day 14–21 at a split ratio of (1:4–1:6) (Fig. 1c). Using the optimised medium, hPOs can be robustly expanded up to at least 6 months (Fig. 1d,e, Additional file 1: Figure S1a). To date, we have derived hPO lines capable of long-term expansion from 27 out of 29 healthy human donors (i.e. donors without any known pancreatic disease) with an age range of 24–79 years. Of note, successful hPO establishment is independent of the donor’s sex, age or BMI (Additional file 2: Table S1), while unsuccessful isolations were due to technical reasons.

﻿While our work was ongoing, a report [22] described culture conditions that support hPO generation with a similar efficiency to our optimised media (hPO-Opt.EM) (Additional file 1: Figure S1b). However, as with previous studies, the culture medium reported [22] does not efficiently sustain long-term expansion of hPOs, as they deteriorate rapidly from P3–4 (Additional file 1: Figure S1c,d). Using hPO-Opt.EM, the hPOs can be expanded with high efficiency, exhibiting an initial doubling time of 78 h which slows to 177 h at later passages (Fig. 1f). These data indicate that hPOs are capable of the vast expansion required for both, disease modelling as well as cellular therapies.

A key benefit of many recent organoid systems is the ability to cryopreserve organoids at early passages and then thaw at later timepoints to re-establish cultures. As expected, the hPO cultures reported here are amenable to cryopreservation and expandable cultures can be readily re-established upon thawing (Fig. 1g). The ability to cryopreserve hPO cultures enables the sharing and storage of resources, yet it still requires hPO derivation to be conducted as quickly as possible following sample collection. By contrast, cryo-banking of primary tissue would further facilitate the workflow for global collection and subsequent distribution of tissue world-wide, including to labs situated at great distances apart. Hence, we next tested whether hPO cultures could be initiated from cryopreserved tissue. We first optimised the cryopreservation of the fresh tissue and subsequent organoid derivation from it. Prior to cryopreservation, the primary tissue was mechanically minced so that upon reconstitution in the freezing medium the tissue would be more uniformly suspended and cool in a more homogeneous manner than one large piece of tissue. The tissue was then stored at − 80 °C for 3 weeks. Upon thawing, tissue fragments were washed to remove any remaining freezing medium and subsequent derivation was performed as with fresh tissue. Organoid derivation efficiency was on average 10-fold lower from cryopreserved tissue compared with fresh tissue (Additional file 1: Figure S2a,b), however, in all cases, hPOs were successfully generated from cryopreserved samples (Fig. 1h, Additional file 1Figure S2a). hPOs generated from cryopreserved tissue displayed similar expansion efficiencies as cultures derived from freshly isolated pancreata (Additional file 1: Figure S2c). Using this methodology, donor material can be collected, cryopreserved and transferred to a recipient laboratory for derivation without time restrictions.

In addition to the expansion and passaging of organoids upon mechanical dissociation of organoid structures into fragments (Additional file 1: Figure S2d), our hPO culture system supports expansion from dissociated single cell suspensions. The colony formation efficiency from single hPO cells does not significantly decrease during long-term culture (Additional file 1: Figure S2e), and therefore single cells can be isolated at both early and late passages and cultured to generate expandable cystic organoids. The ability to expand from single cells opens up opportunities for genetic studies as well as genetic manipulation of the cultures. As an example, we have generated mutant hPOs from single cells following viral infection with a lentivirus containing a GFP reporter. Following viral infection, the cells underwent fluorescent cell sorting (Additional file 1: Figure S2f) to select for successful viral integration and were then expanded to generate GFP positive hPO cultures (Additional file 1: Figure S2 g).

In addition, we have generated clonal cultures for studies of genome integrity by first seeding single cells at low density, picking out newly formed single organoid structures and transferring each organoid to a separate BME 2 drop. The single organoid can then be passaged to generate hPO cultures of the same clonal origin (Fig. 1i,j). Comparison of the mean variant allele frequency (VAF) of single nucleotide variants can be used to assess the clonality of cells. The VAF for three different cultures derived in this manner from the same donor was close to 0.5 (Clone 1 = 0.45, Clone 2 = 0.51, Clone 3 = 0.50), suggesting that each of these hPO cultures was derived from the clonal expansion of a single ductal cell in vitro (Fig. 1k).

In summary, our optimised hPO culture system enables the long-term expansion of human primary ductal pancreas tissue from both fresh and cryopreserved samples, and even as clonal cultures.

### Characterisation of human primary tissue-derived pancreatic organoids

**Fig. 2:**
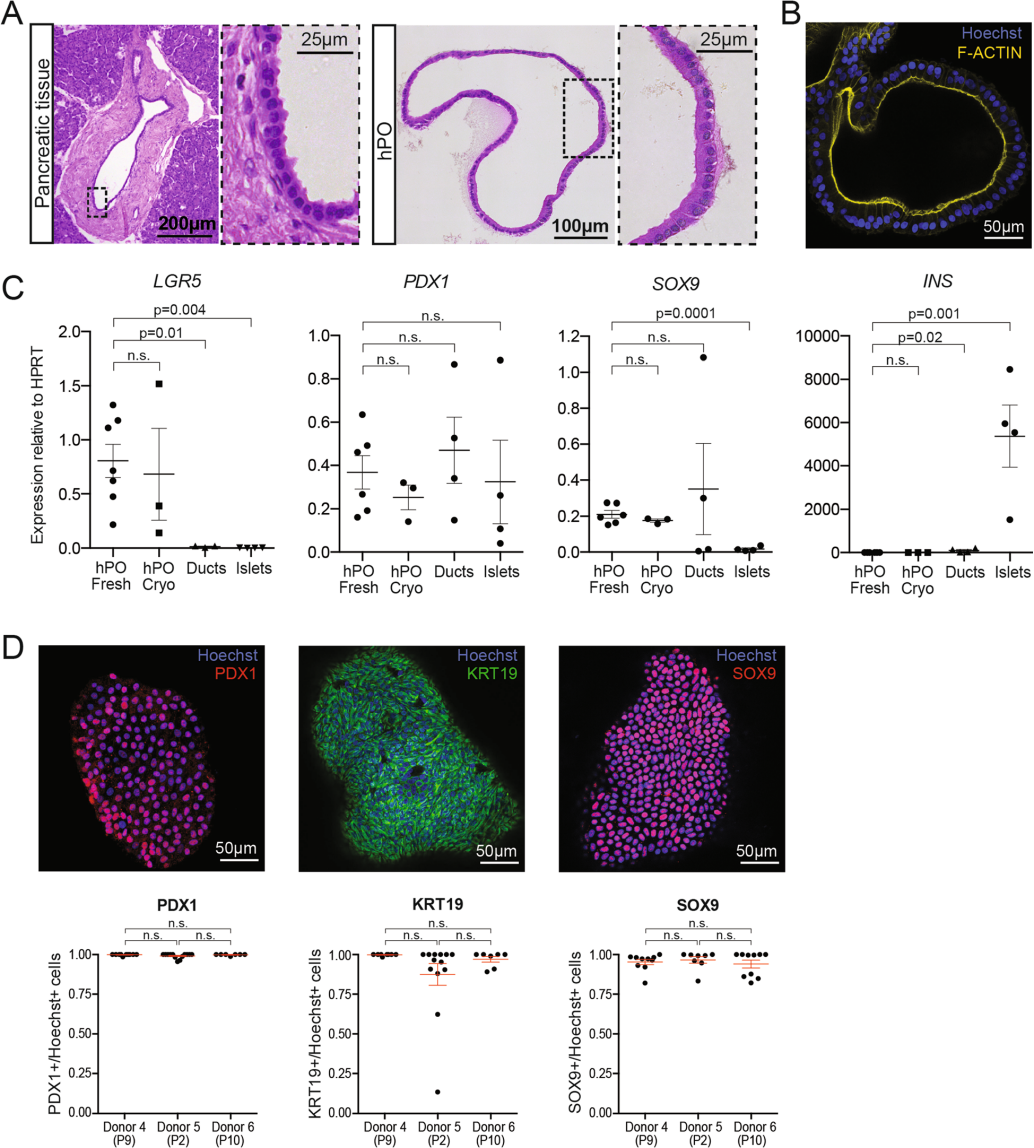
Human pancreatic organoids (hPOs) expanded long-term recapitulate pancreatic ductal epithelium in vitro. **a** Representative images of H&E staining of human pancreatic ductal tissue and hPOs. Note that hPOs (right) expanded in culture retain the single-cell morphology exhibited by the pancreatic ductal tissue in vivo (left) (*n* = 6 independent donors). **b** Representative immunofluorescence staining of F-Actin (yellow) demonstrates that hPOs maintain the epithelial cell polarity typical of ductal tissue (nuclei counterstained with Hoechst, blue) (*n* = 6 independent donors). **c** mRNA expression analysis of key genes involved in stem cell biology (*LGR5*), pancreatic fate (*PDX1*), ductal fate (*SOX9*) and β-cell function (*INS*) in hPOs derived from fresh tissue (hPO Fresh, *n* ≥ 6), cryopreserved tissue (hPO Cryo, *n* = 3), isolated primary ducts (*n* = 4) and isolated islets (*n* = 4). **d** Immunofluorescence staining (upper panel) and quantification of positive cells (lower panels) of nuclear PDX1 (red), cytoplasmic KRT19 (green) and nuclear SOX9 (red) protein in hPOs. Graphs represent number of positive cells for the corresponding marker (≥7 organoids counted per donor). Graphs show mean ± SEM

Pancreatic ducts are single cell-layered structures that are responsible for the collection and transfer of digestive enzymes produced by acinar cells to the duodenum. hPOs recapitulate the single cell-layer morphology and epithelial polarisation of their tissue of origin (Fig. 2a,b). mRNA expression analysis of hPOs (derived from either fresh or cryopreserved tissue), isolated primary ducts and isolated islets (Fig. 2c) reveals that hPOs express increased levels of the adult stem cell marker *LGR5* [25]. hPOs, isolated ducts and islets all express similar levels of the pancreatic progenitor and beta-cell marker *PDX1*. We find hPOs express higher levels of the ductal marker *SOX9* in comparison to islets, whilst there is no significant difference between hPOs and isolated ducts. These findings suggest hPOs maintain a pancreatic ductal identity during in vitro culture. This is further supported as hPOs and isolated ducts express significantly less insulin mRNA than islets. It should be noted that some insulin expression was detected in the ductal preparation. We hypothesise that this is likely due to an artefact of the isolation method used for primary ducts, which although enriches for ductal cells, may also include other contaminating acinar and endocrine tissue. Furthermore, we find hPOs maintain expression of SOX9 and KRT19 (ductal markers) as well as PDX1 at the protein level during long term culture (Fig. 2d), consistent with the preservation of ductal identity over months in culture. Of note, similar expression patterns and tissue architecture were observed from hPOs derived from cryopreserved tissue as compared to hPOs derived from freshly isolated tissue (Figs. 2c and Additional file 1: Figure S3a,b). Therefore, our chemically defined, optimised pancreas organoid medium supports the long-term expansion of human pancreatic tissue as ductal epithelial cells from both fresh and cryopreserved donor tissue (Additional file 1: Figure S2b).

### Genomic stability and in vivo safety of hPOs expanded long-term in culture

**Fig. 3:**
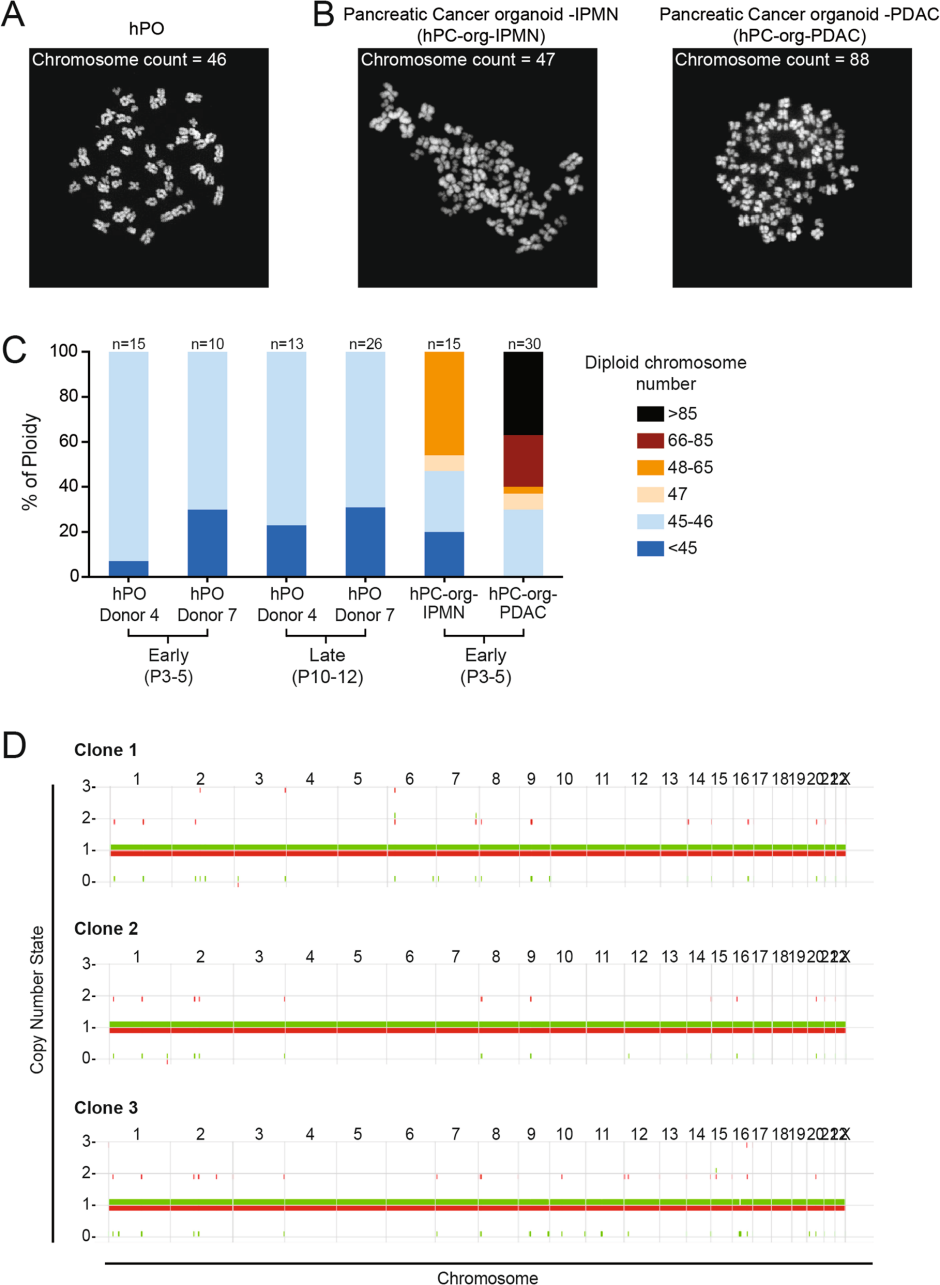
Human pancreatic organoids (hPOs) expanded long-term in culture maintain chromosomal stability over time. **a-b** Representative images of chromosome spreads used for counting from (**a**) healthy human pancreas derived organoids and (**b**) pancreas cancer derived organoids (generated from pancreatic tumour tissue; Intraductal Papillary Mucinous Neoplasm (hPC-org-IPMN) and Pancreatic Ductal Adenocarcinoma (hPC-org-PDAC). **c**) Chromosome spreads were prepared and counted from early (P3–5) and late (P10–12) passage cultures. Note that hPOs generated from healthy donors do not display increased chromosomal counts (above 46) indicating hPOs maintain normal chromosome numbers during in vitro culture, whilst this is not the case for pancreas cancer organoids, as previously reported [21]. The number of chromosome spreads counted per condition is detailed above the graph. D) ASCAT copy number plots of three clonal hPO cultures show that hPOs do not exhibit loss of chromosomes or large structural rearrangements during in vitro culture (clonal expansion of 5 weeks). The copy-number state for each chromosome is shown on the Y-axis, with one allele coloured in red and the other in green. Chromosomes are labelled along the top of the graphs

Somatic mutations and chromosomal abnormalities accumulate spontaneously throughout the lifetime of an individual, and while most are harmless, others act as driver mutations which increase the likelihood of cell transformation and tumorigenesis [26]. For accurate disease modelling as well as use as a cell therapy, it is vital that the cells used do not show an increased susceptibility to accumulate genetic aberrations upon time in culture, which could interfere with the conclusions obtained from the disease model or cause tumour formation in the recipient patient if used as a cell therapy. We analysed the number of chromosomes in both early- and late-passage hPOs to evaluate their genomic stability over time in culture (Fig. 3a). As a positive control, we analysed the number of chromosomes in human pancreatic cancer organoids derived from either Intraductal Papillary Mucinous Neoplasm or Pancreatic Ductal Adenocarcinoma cancerous tissues (hPC-org-IPMN and hPC-org-PDAC, respectively). As expected, we found abnormal chromosomal numbers even at early stages in hPC-org cultures, with more than 50% of the cells exhibiting chromosomal numbers greater than 46, with the most severe chromosomal number changes detected in organoids derived from the more aggressive PDAC tissue (Fig. 3b,c). In contrast we never detected chromosomal numbers greater than 46 in either early- or late-passage hPOs (Fig. 3a,c). In order to assess whether hPOs undergo large-scale chromosomal rearrangements or chromosome loss, we conducted copy-number analysis on clonal cultures. Whole genome sequencing was performed on three independently derived clonal cultures to ~35x depth. The allele-specific copy number analysis of tumours algorithm (ASCAT) [27] was then used to call copy-number changes. ASCAT makes use of both read depth and the ratio of heterozygous SNPs to determine allele-specific copy number. The results reveal no evidence of large-scale chromosomal differences in the organoids (Fig. 3d, Additional file 3**:** Table S2) indicating that large-scale chromosomal defects do not occur as a consequence of long-term in vitro culture (Fig. 3c,d).

For a successful cell therapy, cells need to persist long-term in the body without giving rise to tumorous growths or teratomas. Previous studies that have performed xenografts using healthy human pancreatic organoid cells have reported a low engraftment efficiency (12.5% of xenografts resulted in cells that could be recovered within the 1 month timepoint, and no xenografts recovered at later timepoints) [21]. We therefore tested the ability of our hPOs to engraft and be maintained long-term (beyond the reported 1 month limit) as well as their potential tumorigenicity in vivo. In order to achieve long-term (3 month) engraftment of hPOs in immunodeficient mice, we first tested multiple injection protocols by combining different ECMs, injection sites, as well as the addition of several growth factors and media to act as an injection vehicle (Additional file 4**:** Table S3). We performed injections using Matrigel (a commonly used yet ill-defined xenogenic ECM), BME 2 (a variant of Matrigel with a higher tensile strength and lower batch-to-batch variability) and Glycosil Hyaluronic Acid (G-HA, a chemically defined ECM).

**Fig. 4:**
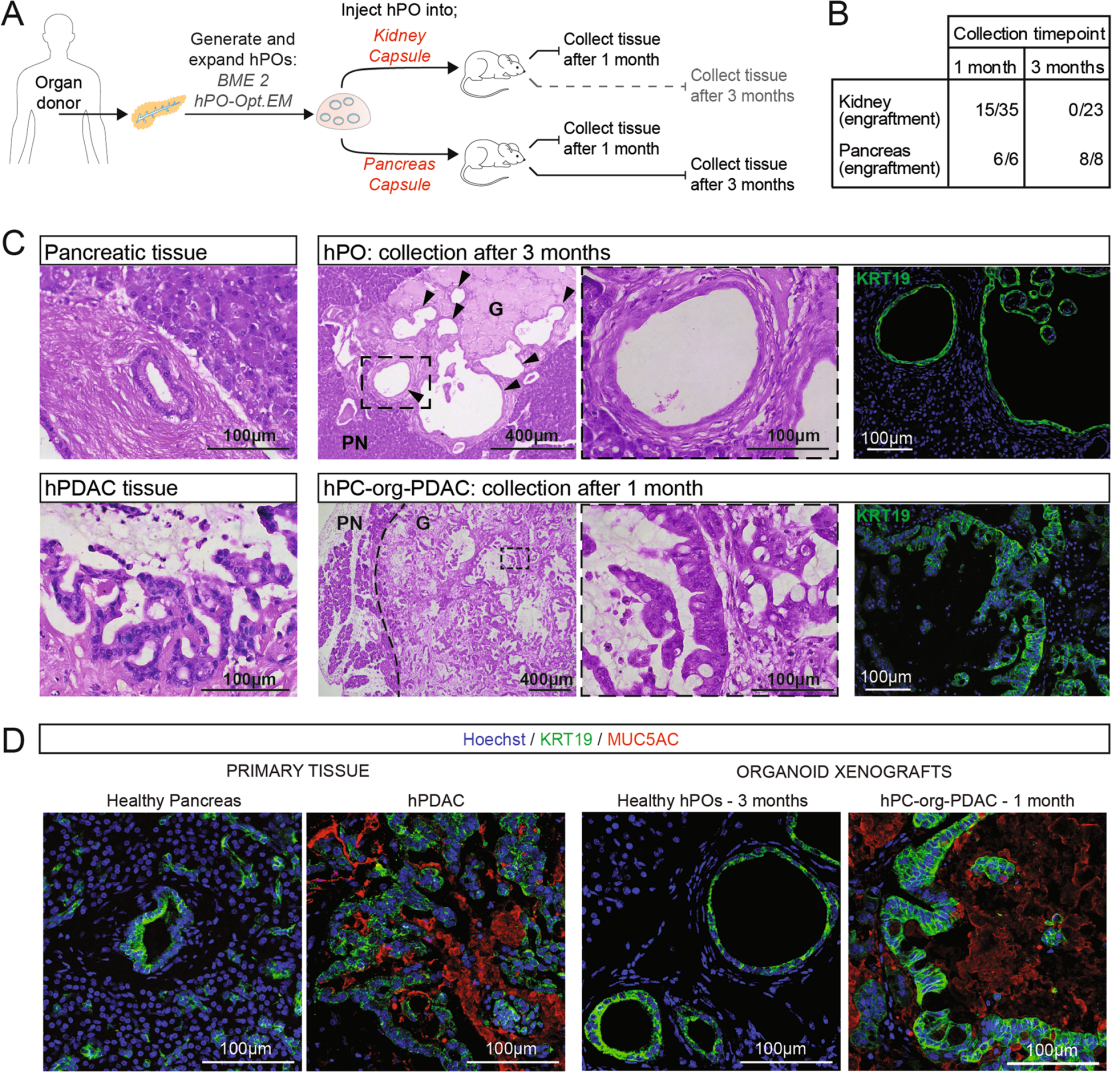
Expanded human pancreatic organoids (hPOs) do not show signs of transformation following long-term engraftment. **a** Experimental design. Following hPO generation and expansion with BME 2 and hPO-Opt.EM, hPOs were transplanted into either the kidney capsule or pancreas capsule of NSG mice; tissues were collected after 1 month or 3 months. **b** Summary of engraftment success after 1 month or 3 months for all hPOs injected, including multiple injection compositions of ECMs and growth factors (for full details please see Table S3 and Fig. S4). **c** H&E staining demonstrates survival of hPOs (G-Graft) after 3 months in the mouse pancreas (PN-pancreas) and shows engrafted hPOs are formed by a single cell-layered epithelium (upper middle panel) recapitulating the ductal tissue structure of a healthy pancreatic tissue (upper left panel). Xenografts of pancreas cancer organoids (hPC-org-PDAC) obtained after 1 month resulted in aberrant ductal morphology reminiscent of the tumour of origin (lower panels), as expected. The human origin of the engrafted cells in the mouse pancreas is confirmed by expression of human-specific KRT19 (green), nuclei counterstained with Hoechst (blue) (right panels). **d** Analysis of primary tissue shows expression of the cancer marker MUC5AC (red) only in tissue from a PDAC tumour resection and not in healthy tissue (*n* = 4). Of note, MUC5AC is absent in xenografts from organoids derived from healthy donors (*n* = 4), even at 3 months, while it is strongly expressed in xenografts derived from hPC-org-PDAC organoids already after 1 month (*n* = 2)

We had previously found that subcutaneous injections of healthy ductal organoids do not result in engraftment (data not shown), and therefore we decided to test the more vascularised kidney capsule and pancreas capsule as potential injection sites (Fig. 4a). Following several iterations, we found that the site of injection had a clear effect on engraftment success. Initial attempts to engraft hPO cells using 100% BME 2 in the kidney capsule resulted in poor engraftment (20% of animals) within the 1 month timepoint (Additional file 1: Figure S4a; K-0). Addition of VEGF, Rho Kinase inhibitor and hPO-Opt.EM medium as a vehicle for the cells, as well as a dilution of the ECM to 30%, improved engraftment efficiency after 1 month in the kidney capsule (56%; 5 out of 9 mice) (Additional file 1: Figure S4a and S4b; K-1,K-2,K-3). Remarkably, we found that injection to the pancreas caspule allowed 100% engraftment at 1 month regardless of ECM dilution or vehicle supplementation with VEGF and hPO-Opt.EM medium (Additional file 1: Figure S4a and b; P-1,P-2). This led us to further test the ability of the pancreatic niche to support hPO cell survival at 3 months; we observed 100% engraftment of cells when using either Matrigel or the chemically defined G-HA. Overall, transplants of hPO cells into the pancreas capsule resulted in 100% engraftment at 1 and 3 months (6/6 and 8/8 mice, respectively) while only 43% of transplants into the kidney capsule could be recovered at 1 month (15 out of 35 mice, Additional file 4**:** Table S3) with none surviving past the 1 month timepoint (0 out of 23 mice) (Figs. 4b, Additional file 1: Figure S4a). Together these results indicate that mixing cells with growth factors, dilution of the ECM and injection to the pancreatic niche have a positive effect on the engraftment capability of hPOs.

We then used our optimised xenograft method (injection of cells with a vehicle supplemented with growth factors in 30% Matrigel into the pancreas) to test whether the engrafted hPOs are indeed derived from the healthy ductal expanded epithelium or represent a potential sub-population of transformed cells that have expanded in culture. Healthy pancreas tissue either does not express or weakly expresses mucins, whereas pancreatic cancer is associated with the overexpression of mucins, for example MUC5AC which is uniquely expressed in pancreatic cancer and used as a diagnostic marker [28]. Xenografts of hPOs do not exhibit cellular transformation at 1 month nor at the latest timepoint assessed (3 months). The engrafted hPO cells display ductal morphology, maintaining the single cell-layered, epithelial organisation characteristic of healthy pancreas ductal tissue and retain expression of KRT19 (Fig. 4c) and SOX9 (Additional file 1: Figure S4c). Furthermore, after 3 months the engrafted cells do not express the cancer-specific mucin, MUC5AC (Fig. 4d). As a positive control we performed xenografts of human cancer hPC-org-PDAC organoids. Injection of hPC-org-PDAC resulted in engraftment and subsequent generation of neoplastic tissue, with ductal cells organised into PanIN structures reminiscent in morphology of the original PDAC tumour (Fig. 4c). As expected and as previously reported for transplanted PDAC-derived organoids [21], we detected the expression of MUC5AC in the engrafted cells (Fig. 4d). Thus, the morphology of the engrafted cells and lack of pancreatic cancer markers indicate that hPOs do not undergo neoplastic transformation in vivo.

### Culture of hPOs with a chemically defined, biomimetic, ECM

The ability of hPOs to undergo expansion and maintain genomic stability both in vitro and in vivo makes them a promising platform for use in basic cellular studies of human pancreas biology, disease modelling and as a cellular therapy. A vital requirement for in vitro studies focused on understanding basic principles of human cellular biology (e.g. cell-cell communication, role of mechanical and physical forces or intracellular communication) as well as disease modelling and cell therapy is the ability to manipulate not only the cells and the medium, but also the ECM in which the cells are cultured. Furthermore, a requirement for a potential cell therapy is the ability to expand cells under GMP conditions. However, the majority of current organoid protocols use Matrigel and BME 2, which cannot be manipulated to change their chemical and/or physical properties (e.g. stiffness). Additionally, these matrices are not appropriate for clinical use due to their xenogeneic origin (Engelbreth–Holm–Swarm mouse sarcoma), which risks pathogenic contamination and immunogenicity complications, as well as batch-to-batch differences, hindering scalability and reproducibility [29]. Hence, we aimed to develop a chemically defined, adjustable, scalable and biomimetic hydrogel in which hPOs could be established and expanded.

**Fig. 5:**
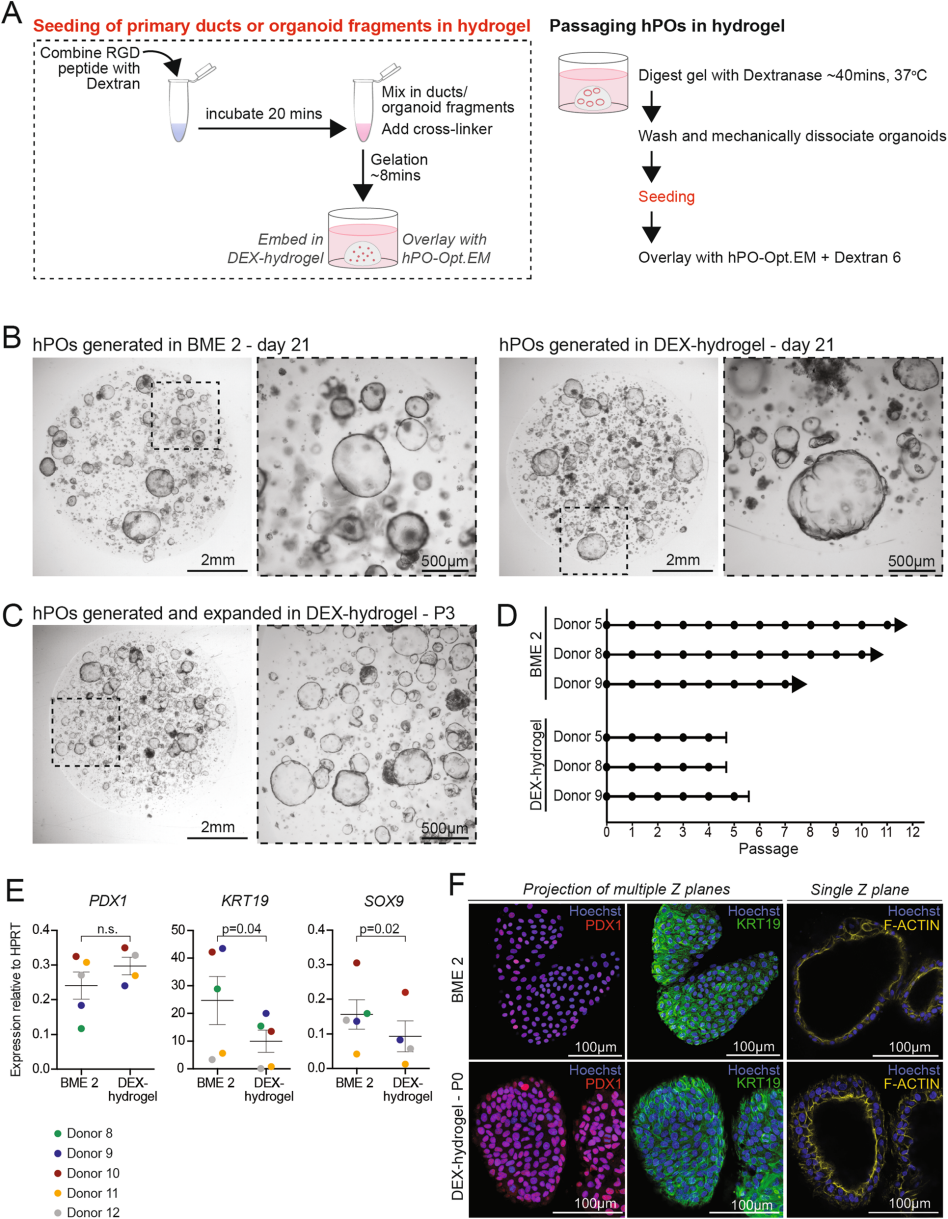
A chemically defined Dextran-based hydrogel supports hPO growth. Organoids were derived and expanded from freshly isolated human pancreas tissue either in standard BME 2 as ECM or in the chemically defined dextran-based hydrogel (DEX-hydrogel). **a** Schematic showing the workflow to use DEX-hydrogel as ECM when seeding ductal fragments for hPO culture initiation or organoid fragments during passaging (left panel). During passaging, dextranase is used to digest the hydrogel and Dextran 6 is added to the culture medium thereafter to prevent hydrogel breakdown (right panel), see methods for details. **b** Representative images of hPO cultures derived from freshly isolated human pancreas tissue and initiated in BME 2 (left) or DEX-hydrogel (right). Pictures were taken 21 days after seeding. **c-d** hPOs can be passaged up to passage 4 when cultured in DEX-hydrogel. Note that, hPOs in DEX-hydrogel expand to a lesser extent than those with BME 2 and cultures begin to deteriorate after P4. **c** Representative images of hPOs in DEX-hydrogel at P3 (*n* = 3). **d** Graph represents the expansion potential of independent donors cultured with BME 2 or DEX-hydrogel. (circle = passage, arrows indicate ongoing cultures, capped lines indicate cultures that deteriorated). **e** mRNA expression analysis of hPO cultures (P1-P4) reveals that organoids grown with DEX-hydrogel retain the expression of ductal and pancreatic genes although *KRT19* and *SOX9* are at a lower level than those cultured with BME 2 (Statistical analysis with paired t-test). **f** Immunofluorescence staining reveals normal cellular polarisation of hPOs in DEX-hydrogel and that the protein expression of ductal and pancreatic markers is maintained in DEX-hydrogel compared to BME 2 (F-Actin - yellow; PDX1 - red; KRT19 - green; Nuclei were counterstained with Hoechst - blue). Experiments were performed in *n* = 2 independent donors

We found that dextran polymers modified with a peptide containing the RGD cell adhesion motif covalently crosslinked with hyaluronic acid in the presence of organoid fragments, in a cell-compatible chemical reaction, supported organoid formation and maintained the epithelial morphology of the organoids (Fig. 5a). Hyaluronic acid was chosen as a crosslinker because it has been shown to support the undifferentiated state of human embryonic stem cells in vitro [30]. Importantly, this hydrogel (DEX-hydrogel) is amenable to digestion by Dextranase, which facilitates passaging and expansion of the hPOs (Fig. 5a). Isolated pancreatic ducts seeded in DEX-hydrogel and cultured with hPO-Opt.EM gave rise to hPO structures in a comparable manner to BME 2 (Fig. 5b). We were able to expand hPOs and perform several passages (up to P4) in the DEX-hydrogel culture system (Fig. 5c,d, Additional file 1: Figure S5a), yet the these organoids expand more slowly and can only be passaged at smaller ratios (1:2–1:3; Additional file 1: Figure S5c) when compared to BME 2 grown hPOs. Characterisation of hPOs generated with either DEX-hydrogel or BME 2 showed that both ECMs supported hPO cultures that expressed similar levels of *PDX1* mRNA (Fig. 5e), expressed PDX1 and KRT19 protein and exhibited similar cell polarisation (Fig. 5f). Of note, the expression of *KRT19* and *SOX9* mRNA in hPOs cultured with DEX-hydrogel was 2-fold lower than in hPOs grown in BME 2 (Fig. 5e), potentially underlying the reduced expansion capacity seen with the DEX-hydrogel. Notably, this chemically defined DEX-hydrogel did not support expansion of hPOs when using a previously published medium [22], with initially formed organoids deteriorating 14 days after seeding and before the structures could be passaged (Additional file 1: Figure S5b). In summary, we have developed a chemically defined, tuneable, reproducible and scalable biomimetic hydrogel which supports hPO growth and initial expansion although long-term expansion is yet to be achieved.

## Discussion

The human pancreas is a complex organ performing a variety of diverse tissue functions, from food digestion to control of glucose homeostasis. This complexity is mirrored in the wide array of pancreas diseases which range from pancreatits to diabetes to pancreatic cancer. Recapitulating healthy and diseased pancreas tissue in vitro has proven challenging due to the lack of robust culture methods that would enable the expansion of non-transformed human pancreas cells while preserving tissue of origin (healthy or diseased) characteristics. Recently, organoid culture systems have emerged as a promising technology to bridge the gap between cell lines and in vivo tissue [31]. Pancreas organoids derived from adult mouse pancreatic ducts recapitulate the ductal epithelium structure and physiology in culture. However, healthy human pancreas tissue has proven more challenging to recreate and expand in culture. While three protocols, including ours, describe the establishment of human pancreatic ductal organoids in vitro [21–23], these suffer from a limited capacity for expansion, use of serum-containing, chemically ill-defined medium and an inability to generate organoids from single cells. These limitations hamper their use in studies of pancreas duct cell biology and genetics as well as for disease modelling of the exocrine compartment and potential cell therapy approaches.

While healthy, non-transformed, human pancreas tissue had proven difficult to maintain ex vivo, human pancreas organoids derived from tumour tissue [21, 32] or tumour biopsies [33, 34] have already been established and utilised for modelling pancreas cancer in vitro and for identifying drug sensitivities, as these faithfully recapitulate the architecture, transcriptome and mutational landscape of the tumour of origin in a patient-specific manner. However, other exocrine pancreas diseases, such as cystic fibrosis or pancreatitis, have not yet been modelled in vitro due to the lack of a culture system for manipulating primary human ductal epithelium ex vivo.

In this work we describe an optimised culture system to enable the long-term expansion of human pancreas ductal cells as human pancreas organoids (hPOs) in a chemically defined, serum-free medium, which will facilitate the development of disease models derived directly from diseased tissue. Critically, we show that our hPO medium enables the clonal expansion of healthy ductal cells, which had previously proved challenging [4, 19], thus facilitating their genetic manipulation. This holds the potential to promote the development of in vitro models for exocrine diseases following step-wise, guided genetic manipulation, as described for colon cancer, where the sequential addition of mutations using CRISPR technology has enabled the identification of the minimal set of mutations that can induce colon cancer in healthy human colon cells [35]. This opens up unprecedented opportunities for the study of developmental lineages as well as mutational processes in human pancreas tissue, which requires the study of clonally-derived cells, similar to studies performed in murine systems investigating the clonal evolution of colon and stomach cells [18, 36].

The ability to recapitulate exocrine diseases of the ductal compartment in culture, either directly from diseased tissue or after the sequential addition of mutations to healthy epithelium, would enable their use as platforms to identify treatments for these diseases. On that front, there have been advances using PSCs (either ESCs or iPSCs) for disease modelling of exocrine pancreas diseases such as developmental defects [37], pancreas cancer [38] and cystic fibrosis [39]. However, the need for cell reprogramming and subsequent erasure of the cell’s epigenetic memory prevents modelling of the adult disease in full, and is particularly limited in recapitulating the influence of the (epi) genome in terms of disease establishment and progression. Here, we demonstrate that our hPOs maintain chromosome stability and do not undergo transformation during long-term engraftment in vivo, which makes them a promising system for disease modelling as well as the genetic manipulation mentioned above.

One prevalent pancreatic disease that may be treatable following further development of hPOs is Type 1 Diabetes. T1D is caused by a lack of insulin-producing β-cells in the pancreatic islets. The most common treatment is administration of exogenous insulin, but this neither cures T1D nor prevents its long-term complications including heart, kidney and peripheral vascular diseases. Whole pancreas or islet transplantation restores a therapeutic pool of functional β-cells, which take over the precise physiological control of blood glucose levels. Since it is currently not possible to culture and expand pancreas islets in vitro, only fresh islets are used for transplantation. However, donor scarcity and the requirement for life-long immune suppression make this treatment unviable for the majority of patients. Pancreatic organoids generated from mouse ducts retained the ability to differentiate to endocrine lineages; if this can be replicated in the human, these hPOs may serve as the basis for expanding a large number of cells, which can then be differentiated and transplanted into a patient. We demonstrate that hPOs maintain ductal biomarkers, therefore endocrine differentiation of the hPOs will be required in order to act as a cell therapy for T1D and is the subject of current investigations. There is evidence, though, that indicates endocrine differentiation of hPOs could be feasible. Human pancreatic ductal cells have been reported to become insulin positive as part of the adaptive increase in beta cell numbers during pregnancy [40]. Likewise, in vitro*,* there are suggestions that differentiated ductal cells can be reprogrammed to insulin positive fates, both in mouse [41] and in human cells [42, 43]. For a cell therapy, it would be of great benefit to differentiate the cells without using adenoviral overexpression of transcription factors, which could cause genomic alterations. To that end, Loomans and colleagues have reported a method to prime human pancreatic ductal cells for differentiation during in vitro culture by modulation of the culture medium. Upon transplantation into the kidney capsule, these cells could then further differentiate into insulin+ cells; however, the cells could not delaminate from the ductal epithelium to form bona fide pancreas islets and whether these cells are glucose responsive remains to be determined [22].

Another important aspect in tissue and disease modelling is the ECM, which is a crucial component in all tissues, and for many organoid systems it provides a scaffold and physical sites for cells to attach. Interactions between cells and the ECM have been implicated in many biological processes, including establishment of stem cell niches and cellular differentiation. Additionally, abnormal ECM dynamics are often associated with disease [44]. Recent advances in the generation of in vitro organoid systems with ECM have provided a new opportunity to investigate these interactions; however, the use of ECMs such as Matrigel and BME 2 is suboptimal due to their xenogenic origin, inability to modulate the ECM components and batch-to-batch variability. A fully chemically defined hydrogel has been shown to be able to support growth of human intestinal organoids [45] but as yet, fully chemically defined hydrogels have not been able to support human liver or pancreas organoid culture [46]. Here, as a proof-of-principle, we present a fully chemically defined ECM that can support hPO derivation and culture with a similar morphology compared to organoids grown in BME 2, and which therefore could provide a starting point for future biochemical and physical cell-ECM studies. Due to its chemically defined nature, it also has advantages over Matrigel and BME 2 in developing a GMP compliant production procedure. Additionally, the possibility of manipulating the ECM composition might facilitate the in vitro modelling of the pathogenic ECM-remodeling often observed in pancreatic diseases ranging from pancreatitis to pancreatic cancer. It should be noted, though, that the hPOs expand much more slowly in our defined ECM than in BME 2 (Additional file 1: Figure S5c). Therefore, optimisation of the chemically defined hydrogel for long-term expansion of hPOs is still to be achieved and will be the subject of future studies.

Our work demonstrates a robust model for the expansion of human pancreatic ductal cells as hPOs, which maintain cell identity as well as genomic integrity. This work will pave the way for further studies of epithelial biology, pancreatic disease modelling, as well as providing a potential source for a novel diabetes treatment.

## Conclusions

hPOs can be expanded long-term, from both fresh and cryopreserved human pancreas tissue in a chemically defined, serum-free medium with no detectable tumorigenicity. hPOs can be clonally expanded, genetically manipulated and are amenable to culture in a chemically defined hydrogel. hPOs therefore represent an abundant source of pancreas ductal cells that retain the characteristics of the tissue-of-origin, opening up avenues for modelling diseases of the ductal epithelium and increasing understanding of human pancreas exocrine biology as well as for producing insulin-secreting cells for the treatment of diabetes.

## Methods

### Primary human tissue

Primary pancreas tissue was obtained by the Cambridge Biorepository of Translational Medicine (CBTM) from deceased organ donors from whom multiple organs were being retrieved for transplantation. Pancreas samples were taken via two routes: from donors during the organ retrieval operation (in which organs other than the pancreas were taken for transplant) or from pancreata which were initially removed for organ transplantation but were subsequently declined and allocated for research. Tissue samples were placed in cold University of Wisconsin organ preservation solution prior to transportation to the laboratory.

Donor tissue was taken after obtaining written informed consent from the donor’s family for studies approved by the NRES Committee East of England, Cambridge South for the Department of Surgery, University of Cambridge, REC reference; 15/EE/0152 and the NRES Committee East of England - Cambridgeshire and Hertfordshire Research Ethics Committee for the Department of Surgery, University of Cambridge, REC reference; 16/EE/0227. Pancreas cancer tissue was obtained from patients undergoing pancreatic resection surgery who had given full written informed consent for studies approved by the NRES Cambridgeshire 2 Research Ethics Committee for Human Research Tissue Bank, Addenbrooke’s Hospital, REC reference; 11/EE/0011 and NRES Committee London - Westminster Research Ethics Committee for the Department of Surgery, University of Cambridge, REC reference; 15/LO/0753. Samples were taken by clinical histopathologists after gross examination of the resected tissue. Pancreatic islets were obtained from the Scottish National Blood Transfusion Service (SNBTS) Islet Isolation Center (NRES West Midlands- South Birmingham Research Ethics Committee, REC reference; 16/WM/0093).

Isolated primary pancreatic ducts for qRT-PCR analysis were collected via two methods: either by manual handpicking of ductal fragments following pancreas tissue digestion (as detailed below) or via surgical dissection of the main pancreatic duct from the pancreata allocated for research. Briefly, the common bile duct was separated from surrounding tissue and followed towards the ampulla of Vater where it connects to the primary pancreatic duct. The primary pancreatic duct was then separated from surrounding tissue and a segment was isolated.

### Generation and culture of hPO

Handling and processing of samples was performed according to HTA guidelines. To generate organoid cultures, approximately 5 mg of pancreas sample was manually minced and further dissociated with the gentleMACS dissociator (Miltenyi Biotec) for a total of 2 min. Minced tissue was washed twice in Wash medium [Dulbecco’s Modified Eagle Medium (DMEM), high glucose, GlutaMAX, pyruvate supplemented (Life Technologies) with 1% Foetal Bovine Serum (FBS) (Life Technologies) and 1% Penicillin/Streptomycin (10,000 U/mL) (Life Technologies)] and digested in 40 mL of Digestion solution [Collagenase Type I (Sigma-Aldrich) and Dispase II (Life Technologies) at a concentration of 0.125 mg/mL in DMEM containing 0.1 mg/mL DNase I (Sigma-Aldrich)] and placed at 37 °C for 1 to 2 h. Isolated ducts were either hand-picked with a pipette or the whole digestion mixture was filtered with a 100 μm ﻿pore nylon cell strainer (Falcon). Ductal fragments were washed in Basal medium [Advanced DMEM/F12 (Life Technologies) supplemented with 1% Penicillin/Streptomycin, 1% Glutamax 100x (Life Technologies), and Hepes (Life Technologies) 10 mM] and spun at 200 g for 5 min. The cell pellet was mixed with reduced growth factor BME 2-RGF (Basement Membrane Extract Type 2 3533-010-02; AMSBIO, Cultrex), seeded in a 24 well plate and overlayed with the optimised hPO expansion medium (hPO-Opt.EM), unless specified otherwise. BME 2-RGF was used as ECM for all experiments except for those specified in Fig. 5 and Additional file 1: Figure S5 in which hPOs generated in BME 2-RGF were compared with those cultured in the chemically defined hydrogel. hPO-Opt.EM composition: [Basal medium (described above) supplemented with 1X N2 and 1X B27 (both from GIBCO), 1.25 mM N-Acetylcysteine (Sigma-Aldrich), 10% RSPO1 conditioned serum-free media (homemade as previously described [23]), 10 nM [Leu^15^]-Gastrin I human (Sigma-Aldrich), 50 ng/mL EGF (Peprotech), 25 ng/mL Noggin (Peprotech), 100 ng/mL FGF10 (Peprotech), 10 mM Nicotinamide (Sigma-Aldrich), 5 μM A83.01 (Tocris), 10 μM FSK (Tocris) and 3 μM PGE2 (Tocris)]. hPO-Opt.EM was supplemented with 10 μM Rho Kinase inhibitor (Y27632, Sigma-Aldrich) during the first 7 days. After 14 days, passaging was performed as previously described [23]. Cryopreservation of established organoids was conducted as previously described [23].

### Cryopreservation of pancreas tissue

Samples were manually minced and further dissociated using the gentleMACs dissociator (Miltenyi) for a total of 2 min. Samples were resuspended in 1 mL Recovery Freezing medium [(Dulbecco’s Modified Eagle Medium (High Glucose), fetal bovine serum, and DMSO (10%);Gibco)] and cryopreserved in a Cell freezing container at -80 °C. To initiate hPO generation after cryopreservation, the sample was thawed at 37 °C and washed twice in Wash medium. The procedure was then conducted as described above, beginning with the addition of Digestion solution.

### Dispersion of hPOs to single cells

Preparation of single cell mixtures was performed as previously described [23]; briefly, confluent hPO wells were collected and washed with cold Basal medium. Cells were centrifuged at 200 g for 5 min and resuspended in 1 mL of pre-warmed trypsin (TrypLE™ Express Enzyme (1X)-Thermofisher). Organoids were pipetted using a narrowed Pasteur 10 times and incubated for 5 min at 37 °C to make single cells. After incubation, the cells were pipetted 10 times and checked for single cells. Digestion was stopped by adding cold Basal medium and the digest was filtered through a 40 μm ﻿pore nylon cell strainer (Falcon) to remove doublets.

### Doubling time calculation

Doubling time was calculated as follows; the hPO cultures were dissociated into single cells as described above. Cell numbers were counted by trypan blue exclusion at the indicated time points. From the basic formula of the exponential curve y(t) = y0 x e (growth rate x t) (y = cell numbers at final time point; y0 = cell numbers at initial time point; t = time) the growth rate was derived. The doubling time was calculated as doubling time = ln (2)/growth rate for each time window analysed.

### Lentiviral transduction and flow cytometry sorting

hPOs were expanded to passage 3, after which organoids were made into single cells as described above. 1 × 10^5^ cells were resuspended in virus infection medium containing [CMV-GFP-T2A-Luciferase pre-packaged virus (Systems Bioscience, Cat. No. BLIV101VA-1) at a multiplicity of infection (MOI) of 5 (5x10^5^u/μl) with 1:200 TransDux (System Bioscience) and 1:5 MAX Enhancer (Systems Bioscience) with hPO-Opt.EM]. The cell suspension was added to a 24 well plate, spun at 32 °C at 600 g for 10 min and then incubated at 37 °C for 6 h. Cells were then transferred to a 15 mL centrifuge tube, washed twice with Basal Medium and seeded in a 48 well plate with BME 2 and overlayed with hPO-Opt.EM supplemented with Rho Kinase inhibitor.

After 2 passages, cells were again subjected to a single cell dissociation as described above. Single cell preparations, along with negative controls (non-transduced hPOs) were sorted using a MoFlo cell sorter. GFP+ cells were seeded into 48 well plates with BME 2 and hPO-Opt.EM medium (with Rho Kinase inhibitor for the first 7 days). Organoids were expanded for 2 passages and imaged with the Evos Fl Imaging system (Thermofisher) for expression of GFP.

### Generation of clonal cultures

hPO cultures were initiated from ductal fragments as described above, and allowed to grow for 10–14 days. P0 hPOs were made into single cells as described above. Single cells were centrifuged at 500 g for 5 min, re-suspended in BME 2 and then seeded in a 48 well plate at a density of 300–500 cells/well and allowed to expand for 15–20 days. Individual organoids were then picked out and reseeded (1 organoid per BME 2 drop). The single organoid was allowed to expand and then passaged as normal [23]. For WGS 2–4 confluent wells (of a 24-well plate) were collected for each clone, snap frozen in PBS and submitted for genome sequencing.

### Genome sequencing

Cells were lysed using a commercially available kit (Arcturus PicoPure DNA extraction kit; Thermo Fisher Scientific, ca. No. KIT0103). The samples were then sent for library construction and paired-end whole genome sequencing (150 bp) using Illumina XTEN® machines. Sequences were aligned to the human reference genome (NCBI build 37) using BWA-MEM. This resulted in a read depth of approximately ~35x per sample (see Additional file 3**:** Table S2).

Variant calling was performed using the CaVEMan algorithm [47]. CaVEMan operates using a naive Bayesian classifier to derive the probability of all possible genotypes at each analysed nucleotide. For each sample CaVEMan was run using DNA sequenced from splenocytes from the same donor as a matched normal to identify germline SNPs. CaVEMan requires pre-input copy-number options, which were set to major copy number 5 and minor copy number 2 for normal clones, as we find this maximizes detection sensitivity. After variant calling we applied post-processing filters. We filtered against a panel of unmatched normal samples to remove single-nucleotide polymorphisms (SNPs) commonly present in the population. We also applied two filters designed to remove mapping artefacts associated with BWA-MEM: the median alignment score of reads supporting a mutation should be greater than or equal to 140, and below half of these reads should be clipped. Copy-number changes were called using the allele-specific copy number analysis of tumours (ASCAT) algorithm [27]. The same matched normal sample was used as for calling single nucleotide variants with CaVEMan.

### Culture of hPC

Human pancreas cancer organoids were cultured as previously described in Boj et al. [21]. Briefly, the tumour sample was minced and placed in tumour digestion medium [Collagenase type II (5 mg/mL) made up in tumour organoid culture medium (hPC-EM)]. hPC-EM composition: [Basal medium (described above) supplemented with 1X N2 and 1X B27 (both from GIBCO), 1.25 mM N-Acetylcysteine (Sigma), 10 nM gastrin (Sigma), 50 ng/mL EGF (Peprotech), 40% Wnt3a conditioned medium (homemade), 10% RSPO1 conditioned media (homemade), 100 ng/mL FGF10 (Peprotech), 100 ng/mL Noggin (Peprotech), 10 mM Nicotinamide (Sigma), 0.5 μM A83.01 (Tocris), 1 μM FSK (Tocris) and 10 μM Rho Kinase inhibitor (Y27632, Sigma Aldrich)] and was digested overnight at 37 °C. The digest was spun at 300 g for 5 min and washed in Advanced DMEM/F12. The cell pellet was mixed with reduced growth factor BME 2, seeded in a 24 well plate and overlayed with hPC-EM medium. IPMN-derived tumour organoids were cultured in hPC-EM while PDAC-derived organoids were cultured in hPC-EM with 1 μM PGE2 (Tocris).

### Chemically defined hydrogel culture

For the chemically defined dextran-based hydrogel (DEX-hydrogel) used in Fig. 5 and Additional file 1: Figure S5, SG-Dextran (Cellendes Cat. No. M91–3) and RGD Peptide (Cellendes Cat. No. 09-P-001) were used with a thiol-modified hyaluronic acid cross-linker. Thiol-modified hyaluronic acid was prepared as previously described [48], except that 4-(4,6-dimethoxy-1,3,5-triazin-2-yl)-4-methylmorpholinium chloride (DMTMM, TCI Chemicals) was used instead of N-(3-dimethylaminopropyl)-N′-ethylcarbodiimide hydrochloride (EDC) and N-hydroxysuccinimide (NHS) for attachment of cystamine to hyaluronic acid (Lifecore) with an average molecular weight of 57 kDa [49]. Additionally, tris (2-carboxyethyl) phosphine (TCEP, Sigma-Aldrich, Cat. No. C4706) was used instead of dithiothreitol (DTT) to reduce the disulfide bond of the attached cystamines. Thiol-modified hyaluronic acid was purified by extensive dialysis against phosphate buffer at pH 3–5. This procedure yielded a modification of 12% of the D-glucuronic acid and N-acetyl-D-glucosamine disaccharides of hyaluronic acid with thiol groups as determined with the assay as previously described [50].

For preparation of 50 μl DEX-hydrogel; buffer, water and SG-Dextran of the SG-Dextran Kit (Cellendes Cat. No. M91–3) were used; 3 μl buffer (10x CB (pH 7.2)), 12.5 μl Water, 3.4 μl of SG-Dextran (30 mmol/L thiol-reactive groups) and 2.5 μl of RGD Peptide (20 mmol/L thiol groups) were combined and incubated for 20 min at room temperature. Thereafter, 20 μl organoid fragments were added and hydrogel formation was initiated by adding 8.6 μl of thiol-modified hyaluronic acid (50 nmol of thiol groups). The hydrogel/organoid suspension was seeded into 24 well plate during the 8 min pre-gel period and placed in the 37 °C incubator. 30 min after the initiation of crosslinking, the hydrogels were overlayed with the appropriate culture medium.

Passaging of organoids grown in DEX-hydrogel was achieved by first digesting the hydrogels with Dextranase (Cellendes Cat. No. D10–1) for 30–40 min at 37 °C according to the manufacturer’s recommendations. Once the gel was digested, organoids were fragmented by passing through a syringe with 27ga needle 3–5 times. Organoid fragments were washed 4 times with Basal medium and twice with Basal medium containing 11 mg/mL Dextran 6 (Carl Roth; Cat. No. 7615.1) to remove any Dextranase contamination. After the first passage, hPOs in DEX-hydrogel were cultured with hPO-Opt.EM medium supplemented with 10 mg/mL Dextran 6, acting as a competitive inhibitor to Dextranase, to inhibit gel degradation from leftover contaminating Dextranase; fresh medium was applied every day for 3 days post-passaging.

### Chromosome counting

Chromosome counting of organoid cells was performed as previously described [23]. Briefly, 24 h post-passaging, hPOs were incubated with 0.1 μg/mL KaryoMAX Colcemid solution in PBS (Gibco) for 24 h. hPOs were dissociated into single cells as described above, and were subsequently incubated in 1 mL of 0.075 M KCl (Fisher Chemicals) at 37 °C for 10 min. Cells were then fixed in a solution of 3:1 MeOH:Acetic Acid (VWR Chemicals) which was added dropwise while shaking. After fixation, the solution was dropped onto Superfrost Microscope Slides (VWR) for chromosomes to spread. The slide was then allowed to dry and mounted with Vectashield-Dapi (Vector Laboratories) and a coverslip.

### Quantitative RT-PCR

RNA extraction of all material (organoids and primary tissue) was performed using an RNA extraction kit (Qiagen), and as previously described [23]. A complete list of the primers used can be found in Additional file 5**:** Table S4. All qRT-PCR data is displayed as mean + SEM, with each data point representing a separate donor line. Values are given relative to the expression of the housekeeping gene Hypoxanthine-guanine phosphoribosyltransferase (HPRT).

### Organoid and tissue fixation and paraffin embedding

Organoids were collected from wells, washed with Basal medium and fixed in 10% neutral-buffered formalin (Sigma-Aldrich) for 30–40 min on ice. Primary tissue and xenograft-derived tissue was placed directly into 10% formalin and fixed overnight at room temperature. Organoids and tissue were then embedded in paraffin as follows: samples were dehydrated through a series of graded-ethanol solutions, followed by xylene (Fisher) and finally embedded in paraffin. Sections were cut at 5 μm thickness and were placed at 60 °C for 2–24 h.

### H&E of organoids, primary tissue and xenograft tissue

Paraffin slides were rehydrated with xylene, and then decreasing ethanol concentrations (100–50%) and water. Slides were then immersed in Haematoxylin (Sigma-Aldrich), washed and dehydrated in increasing ethanol concentrations (50–100%), a 10 s wash step of Eosin (Sigma) and finally xylene. The slides were then mounted with DPX mounting solution (Fisher).

### Immunostaining of organoids and tissue sections

Organoids were washed in PBS with 0.05% BSA following formalin fixation as described above. Tissue sections were rehydrated as described above for H&E staining. Following rehydration, slides were washed with PBS and subjected to antigen retrieval by heating to 80 °C in 10 mM Sodium Citrate (Sigma), pH 6 for 20 min. Organoids and sections were incubated with blocking solution [Triton X100 (1% for nuclear antibodies and 0.1% for membrane and cytoplasmic antibodies), 1% BSA, 2% donkey serum] for 2 h at room temperature. Primary antibodies were applied at specified dilutions overnight at 4 °C. Organoids and tissues were washed with PBS, and appropriate secondary antibodies were applied for 2 h at room temperature, washed with PBS and nuclei were counterstained with Hoechst33342 (Molecular Probes, Life Technologies). Please refer to Additional file 5**:** Table S4 for primary and secondary antibody information and dilutions.

### Brightfield and confocal imaging

Brightfield imaging of organoids was performed using a Leica M80 stereo microscope (Leica Microsystems) and a Leica DMIL LED microscope (Leica Microsystems). H&E images were taken using a Leica DM400B LED microscope (Leica Microsystems). Karyotypes and IF staining were imaged using a ﻿SP8 White Light inverted confocal microscope (Leica Microsystems) or with a Leica DMI3000 fluorescent inverted microscope (Leica Microsystems). Optical sections were acquired at 3 μm intervals. Images were acquired with Leica application suite X Software and processed using Fiji.

### Mouse xenograft studies

All animal experiments were performed in accordance with UK Home Office regulations (UK Home Office Project License number PPL 70/8702 and PPL P57643EBB). Immunodeficient NOD.Cg-Prkdc^scid^ Il2rg^tm1Wjl^/SzJ (NSG) mice which lack B, T and NK lymphocytes [51, 52] were bred in-house with food and water available ad libitum pre- and post-procedures. Male and female animals were used, aged approximately 6–8 weeks (average weight 20 g/each). Animals were allocated at random to experimental groups, tissue sections obtained from animals were processed, stained and analysed without reference to the identity of the animal groups.

hPO and hPC-org cultures were expanded in order to inject 5 × 10^5^-1 × 10^6^ cells per mouse. Organoids were mechanically dissociated as described for normal passaging and resuspended in the appropriate injection medium as outlined in Additional file 4**:** Table S3. The cells were loaded into a 250 μL glass gastight syringe (Hamilton) with removable 26ga blunt needles (ESSLAB) for injection into the kidney capsule or custom made 26ga point needles, bevelled at a 60^o^ angle (ESSLAB) for injection into the pancreas capsule. Mice were anesthetised using isoflurane gas and the left side of the abdomen or peritoneal abdomen was shaved and cleaned with disinfectant. During the procedure, the mice were kept under anaesthesia and were kept on heat pads at 37 °C. Injections were performed as described below into the kidney capsule or pancreas capsule. Following the xenograft procedure, all animals were kept alive for either 1 or 3 months after which they were humanely euthanised under terminal anaesthesia by inhalation of isoflurane. Tissue was then retrieved for further histological analysis.

### Kidney capsule injections

An incision of the skin was made near the anatomical position of the kidney, the kidney was localised and a further incision of the abdominal wall was made to expose the kidney. The kidney was gently pushed out of the abdomen and kept wet with sterile saline. A small incision to the kidney capsule was made with a sharp needle, then 20 μL of the organoid suspension was injected under the capsule using the blunt needle syringe. A sterile cotton bud was used to apply pressure to the point of insertion to stop bleeding and prevent cell leakage. The kidney was then gently placed underneath the muscle wall. The muscle wall was sutured first using continuous suturing with 5–0 vicryl sutures and interrupted sutures were used to close the skin layer afterwhich 9 mm autoclip wound clips (Harvard Apparatus) were placed on the skin to keep the sutures intact.

### Pancreas capsule injections

An incision of the skin and abdominal wall was made along the midline of the abdomen to expose the visceral organs. The pancreas was exposed and kept wet with sterile saline. Using a sterile cotton bud for traction, organoids were injected into the tail of the pancreas, through the parenchyma, and placed between the inter-lobular space. The cotton bud was then used to stop leakage by applying pressure for 10–15 s. The pancreas was gently placed back to the correct anatomical position and the abdomen wall and skin were sutured using continuous suturing with 5–0 vicryl sutures.

### Statistical analysis

All summary data are presented as mean ± SEM. Statistical tests were performed using Graphpad Prism software (GraphPad 8.1). Sample size (n) values used for statistical analyses are provided in the relevant figure legends. Student’s two-tailed unpaired t-test (or paired where specified) was performed to assess differences between two groups. When performing a t-test, we assumed normality and equal distribution of variance between groups. Significance was set at *P* < 0.05 for all experiments.

## Supplementary information

**Additional file 1: Figure S1.** Optimisation of hPO-Opt.EM culture medium and its expansion potential compared with published pancreatic organoid culture systems. A) To obtain an optimised medium to support hPO isolation and growth, incremental changes were made to previously published protocols. Expansion graphs show the time hPOs survived in vitro for *n* = 4 donors in mouse pancreatic organoid (mPO) medium [20] supplemented with TGFb inhibitor (top graphs) or in our previously reported medium supplemented with TGFb inhibitor, PGE2 and Wnt-conditioned medium containing 10% serum [23] (middle graphs) or in this new optimised, chemically-defined medium (hPO-Opt.EM) containing TGFb inhibitor, PGE2, FSK and no-FBS (bottom graphs). The ability of the hPOs to expand is indicated by passaging events (cirlces), arrows indicate ongoing cultures, capped lines indicate cultures that deteriorated. While the first two conditions were not able to sustain long-term culture, the hPO-Opt.EM medium demonstrates a much greater expansion potential. B-D) Comparison of the hPO-Opt.EM medium to the chemically-defined hPO medium published by Loomans and colleagues during the course of this project [22]. Although both media enable the initial generation of hPOs, the hPO-Opt.EM medium supports long-term culture to a much greater extent than Medium [22]. B-C) Representative images of hPO cultures derived from fresh pancreas tissue using the the hPO-Opt.EM medium or Medium [22] at B) passage 0 (P0, 8-days post derivation; magnification in lower panels; *n* = 3), or C) at Passage 3 (top) and Passage 4 (bottom) in two independent donors. D) Graph shows the expansion potential of hPOs cultured with the hPO-Opt.EM medium or the medium published by Loomans and colleagues [22] (arrows indicate ongoing cultures, capped lines indicate cultures that deteriorated). **Figure S2.** Human pancreas organoids (hPOs) can be derived from fresh and cryopreserved pancreas tissue and are amenable for genetic manipulation. A) Organoid cultures derived from fresh tissue (left) or tissue cryopreserved at the time of collection (right). The organoid formation efficiency from cryopreserved tissue was reduced, yet in all cases, cultures exhibited similar expansion rate to hPOs derived from fresh tissue. Experiments were performed in *n* = 3 independent donors, with similar outcomes. Representative images are shown. B) Organoid formation from fresh tissue is more efficient than from cryopreserved tissue, the number of organoids formed following the isolation of ductal fragments from either fresh tissue (253 ± 58 organoids; black circles) or cryopreserved tissue (25 ± 3 organoids; blue squares) is shown. Ductal fragments were seeded in a 50 μl BME 2 drop and quantified at P0. Data presented as mean ± SEM. C) hPOs derived from fresh or cryopreserved tissue expand at similar rates (circle = passage). D) Following mechanical dissociation all organoid fragments are capable of forming a new organoid (passage). Representative images of hPO culture during passaging are shown. E) The hPO culture system supports expansion from dissociated single cell suspensions, hPO cultures derived from single cells exhibit similar colony formation efficiency at early as well as late passages (*n* = 4 independent donors). F-G) Genetic manipulation of hPOs. hPOs were dissociated to single cells at passage 3 and transduced with a lentiviral vector carrying a GFP reporter gene. Following viral transduction the single cells formed hPOs and were expanded for 2 passages. F) hPOs were dispersed into single cells again and FACS sorting was used to select for GFP-positivity. The GFP+ cells were isolated and expanded as genetically modified hPOs for a further 2 passages. G) Representative images of genetically modified organoids at passage 8 are shown (*n* = 2). **Figure S3.** hPOs derived from fresh and cryopreserved samples expand as a single cell-layer epithelium of ductal cells and are phenotypically indistinguishable. Comparison of hPOs derived from A) fresh tissue (collected at P3) or B) cryopreserved tissue (collected at P2) from the same donor. Brightfield images (upper panels) indicate that hPO cultures expand efficiently as cystic structures regardless of whether the original tissue is fresh (A) or cryopreserved (B). H&E stainings reveal that hPOs derived from cryopreserved tissue maintain the single cell-layer epithelial architecture seen in the original donor tissue and in hPOs derived from fresh tissue (middle panels). Immunofluorescence stainings demonstrate that hPO cultures maintain ductal identity (KRT19 and SOX9), cellular polarisation (F-Actin) and express PDX1 (lower panels) regardless of the original tissue being fresh (left) or cryopreserved (right). **Figure S4.** Optimisation of hPO transplantation. A-B) hPOs were transplanted into NSG mice at different sites using a combination of different vehicles and ECMs, tissues were retrieved 1 and 3 months after injections. A) Table outlining engraftment conditions and engrafment success rates of hPO xenografts conducted with a combination of injection medium compositions and injection sites in order to achieve long-term survival of hPOs in vivo (NT – not tested). B) Representative H&E images of engrafted hPO cells at 1 month (upper panels) or 3 months (lower panels) show engrafted cells form ductal-like structures in vivo (G-graft, PN-pancreas). C) Xenografts of hPOs into the pancreas collected after 3 months show the engrafted cells retain SOX9 protein expression. **Figure S5** hPOs cultured in optimised hPO-Opt.EM medium grow in a chemically defined hydrogel. A-B) Comparison of hPO cultures in the chemically defined dextran-based hydrogel (DEX-hydrogel) initiated from freshly isolated ducts overlayed with either A) hPO-Opt.EM medium or B) the medium published by Loomans and colleagues [22]. Both media support the formation of cystic structures, however, organoids in Medium [22] quickly deteriorated and could not be passaged. In contrast, organoids formed with the optimised hPO-EM medium could undergo expansion up to P4, after which they deteriorated. C) hPOs generated in hPO-Opt.EM medium expand more slowly in DEX-hydrogel as shown by the longer time taken for the cultures to reach confluency in order to be passaged than hPOs embedded in BME 2 (circles-passage events; arrows indicate ongoing cultures, capped lines indicate cultures that have deteriorated).

**Additional file 2: Table S1.** Donor Demographics and organoid derivation success. Table summarising organ donor information including age range, Body Mass Index (BMI), ischaemic time of tissue, underlying pathology and success of organoid isolation. All healthy tissue was procured from deceased organ donors whose organs were retrieved for transplantation. Diseased tissue was procured from patients having undergone surgical resection. Pancreatic islets were isolated in the Scottish National Blood Transfusion Service (SNBTS) Islet Isolation Centre and were subsequently allocated for research.

**Additional file 3: Table S2.** Whole Genome Sequencing and Copy Number analysis of clonally derived hPO cultures. hPOs were expanded as clonal cultures from the same donor and whole genome sequencing (WGS) was performed on the three individual clonal cultures at a sequencing depth of ~35x. The Allele Specific Copy Number Analysis (ASCAT) algorithm was used to assess clonal number variation in the clonal cultures and determine existence of large structural variations.

**Additional file 4: Table S3.** Transplantation of hPOs and hPC-IPMN and hPC-PDAC organoids in immunodeficient mice. Table summarising information for the healthy and cancer-derived organoid cultures transplanted into NSG mice including donor ID, passage at time of injection, injection vehicle composition (Condition), number of mice transplanted (Mice Tx), location of transplant, overall engraftment success and furthest timepoint of engraftment.

**Additional file 5: Table S4.** Materials used for experiments. Table summarising list of antibodies used for immunostaining, kits used, as well as primers used for quantitative RT-PCR.

## Data Availability

Whole genome sequencing data has been deposited in the European Genome-Phenome Archive under study accession EGAS00001002626.

## Abbreviations

BME 2: Basement Membrane Extract Type 2
ECM: Extracellular Matrix
ESC: Embryonic Stem Cell
FSK: Forskolin
G-HA: Glycosil Hyaluronic Acid
GMP: Good Manufacturing Practice
H&E: Haematoxylin & Eosin
hPC-org: Human Pancreatic Cancer-organoid
hPO: Human Pancreas Organoid
hPO-Opt.EM: Human Pancreas Organoid-Optimised Expansion Medium
IPMN: Intraductal Papillary Mucinous Neoplasm
iPSC: Induced Pluripotent Stem Cell
PDAC: Pancreatic Ductal Adenocarcinoma
PGE2: Prostaglandin E2
RGD: Arg-Gly-Asp
T1D: Type 1 Diabetes
TGFb: Transforming Growth Factor beta
VAF: Variant Allele Frequency
VEGF: Vascular Endothelial Growth Factor
WGS: Whole Genome Sequencing
