# Supplementary material for: Long-term expansion, genomic stability and in vivo safety of adult human pancreas organoids
Source: BMC Dev Biol. 2020 Feb 26;20:4. doi: 10.1186/s12861-020-0209-5 (PMC7043048; doi:10.1186/s12861-020-0209-5)

Supplementary Figure 1 - Optimisation of hPO-Opt.EM culture medium and its expansion potential compared with published pancreatic organoid culture systems.

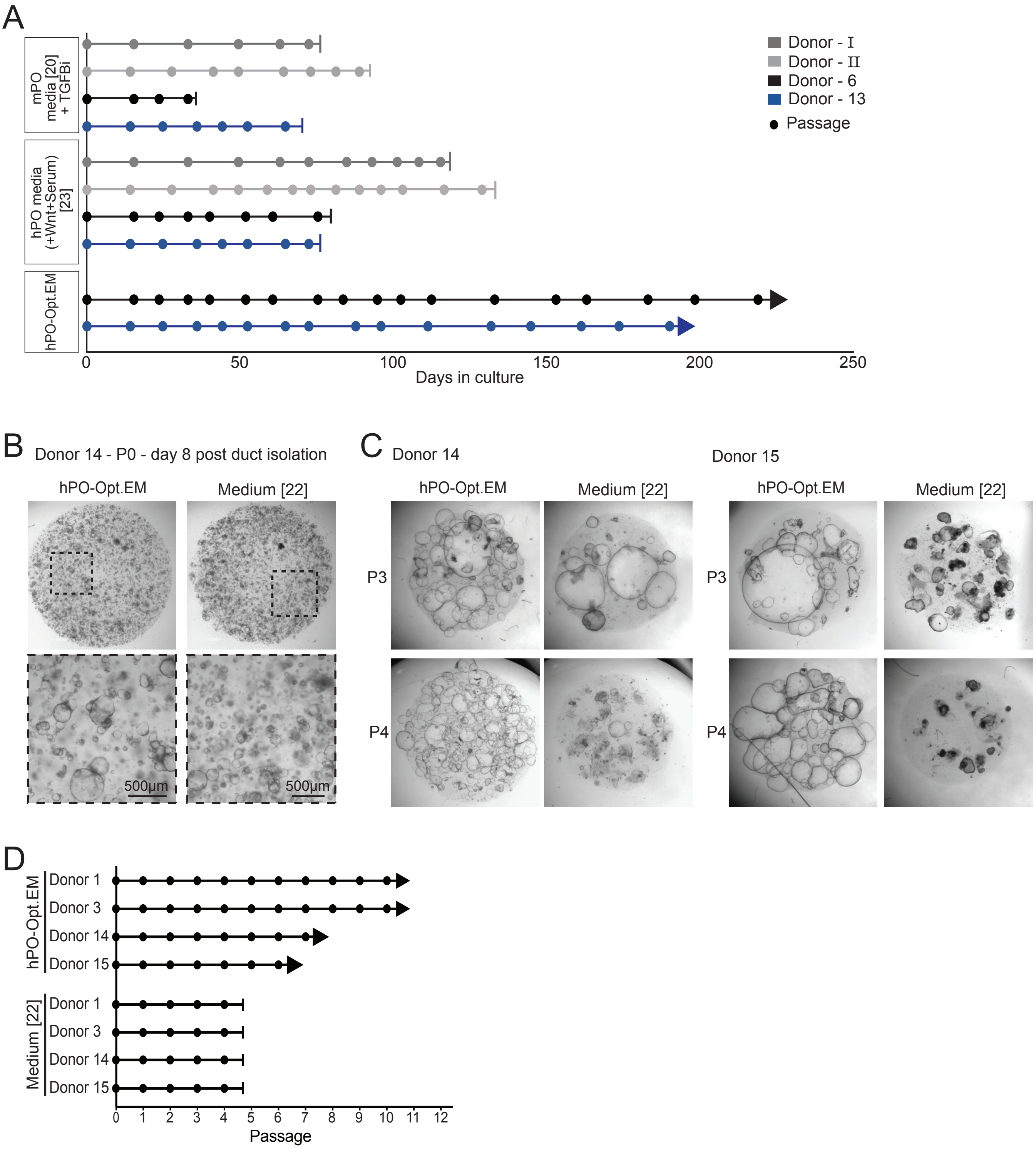

Supplementary Figure 2 - Human pancreas organoids (hPOs) can be derived from fresh and cryopreserved pancreas tissue and are amenable for genetic manipulation.

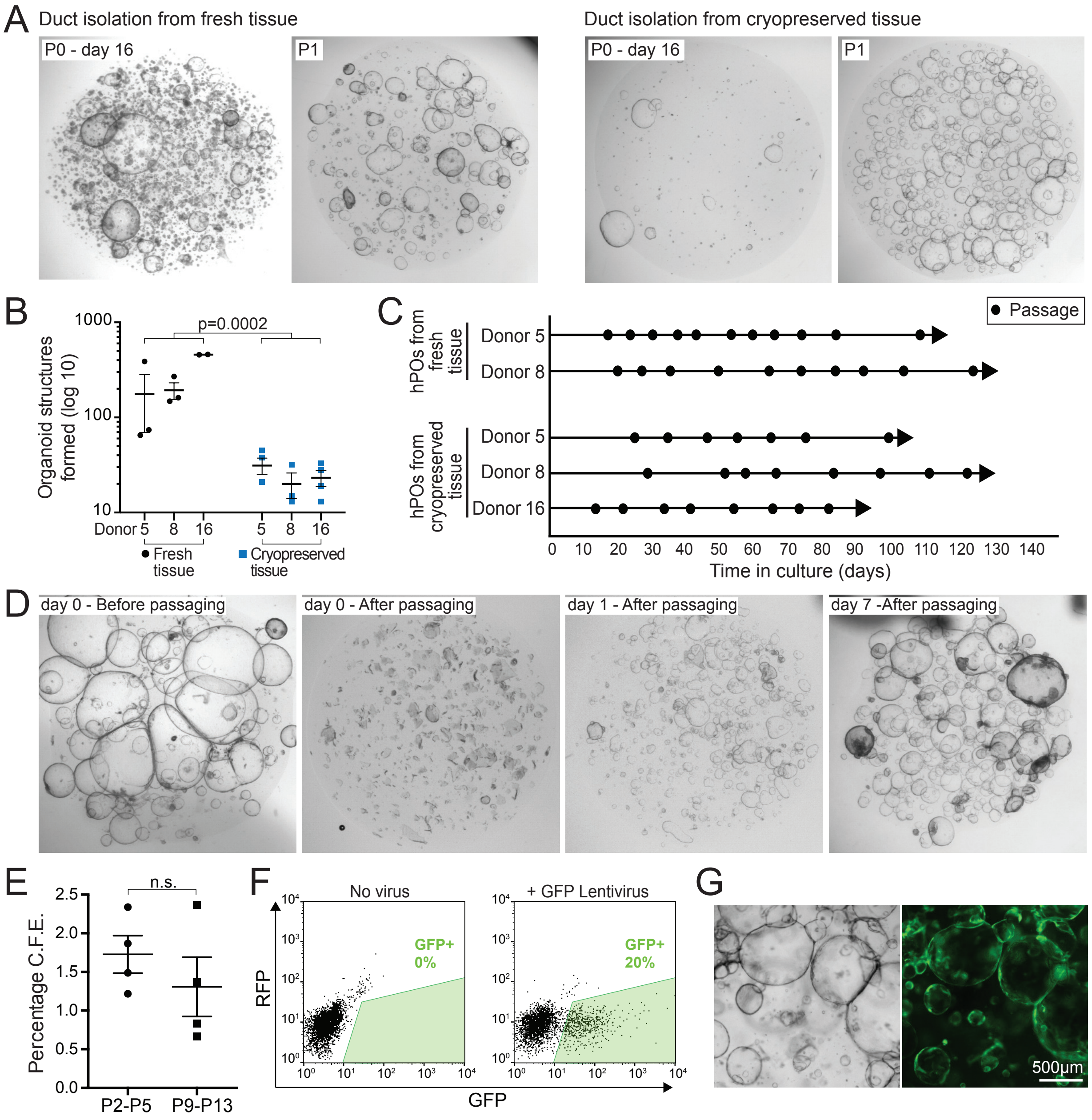

Supplementary Figure 3 - hPOs derived from fresh and cryopreserved samples expand as a single cell-layer epithelium of ductal cells and are phenotypically indistinguishable.

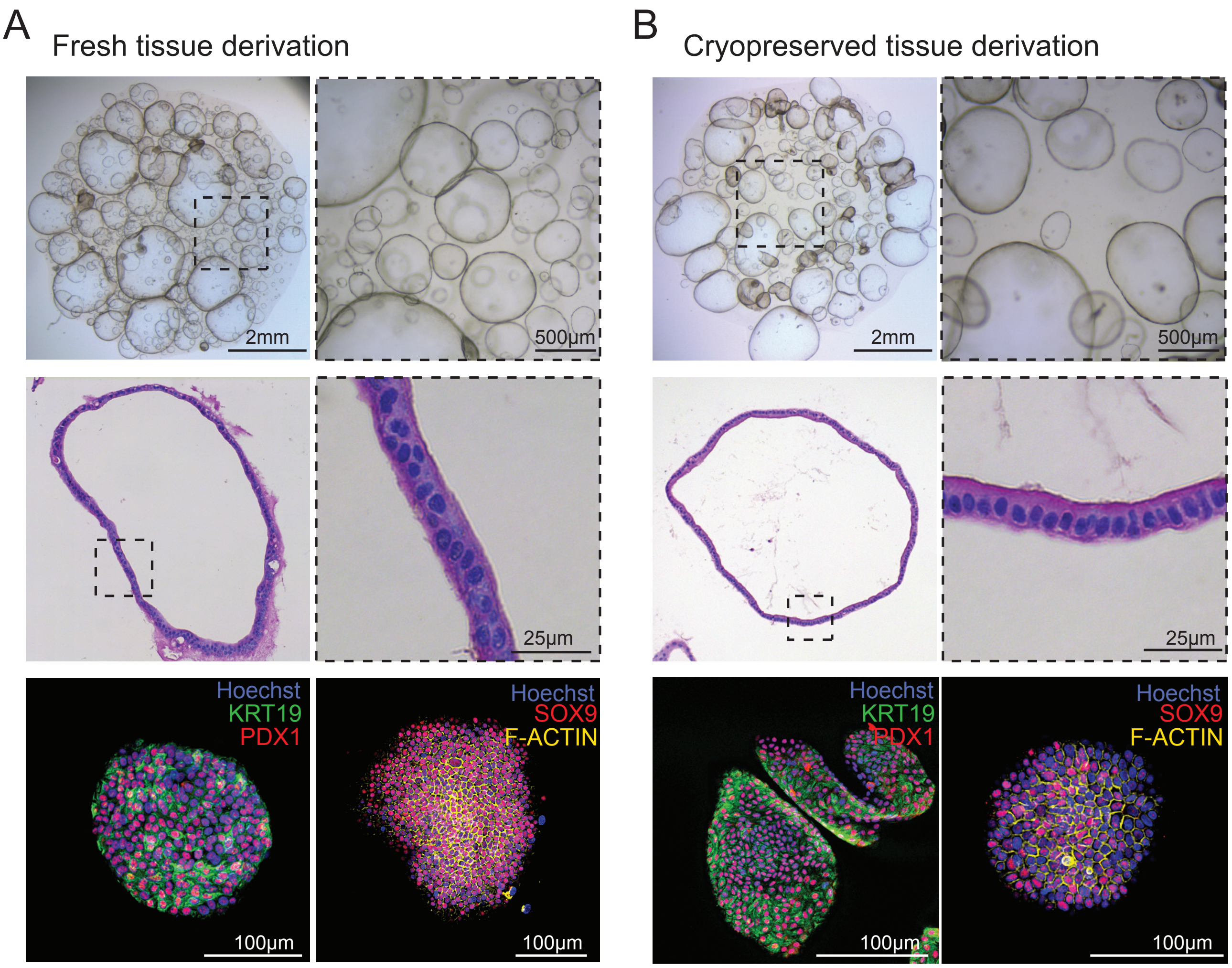

Supplementary Figure 4- Optimisation of hPO transplantation.

A

Condition	K-0	K-1	K-2	P-1	K-3	P-2	P-3	P-4
**Location**	Kidney	Kidney	Kidney	Pancreas	Kidney	Pancreas	Pancreas	Pancreas
**Matrix type**	BME 2	BME 2	BME 2	BME 2	Matrigel	Matrigel	Glycosil Hyaluronic Acid	Matrigel
**Matrix (%)**	100	100	30	100	30	30	50	30
**VEGF**	✗	✗	✓	✗	✓	✗	✓	✓
**Rho Kinase inhibitor**	✗	✓	✓	✓	✓	✓	✓	✓
**hPO-Opt.EM Medium**	✗	✗	✗	✗	✓	✗	✓	✓
**Engraftment Success (1 month)**	4/20	2/3	2/3	3/3	1/3	3/3	1/1	1/1
**Engraftment Success (3 months)**	0/23	NT	NT	NT	NT	NT	3/3	3/3
**Time of *in vitro* expansion**	35 mice- 60 days<br>8 mice- 135 days	60 days			60 days		40 days	

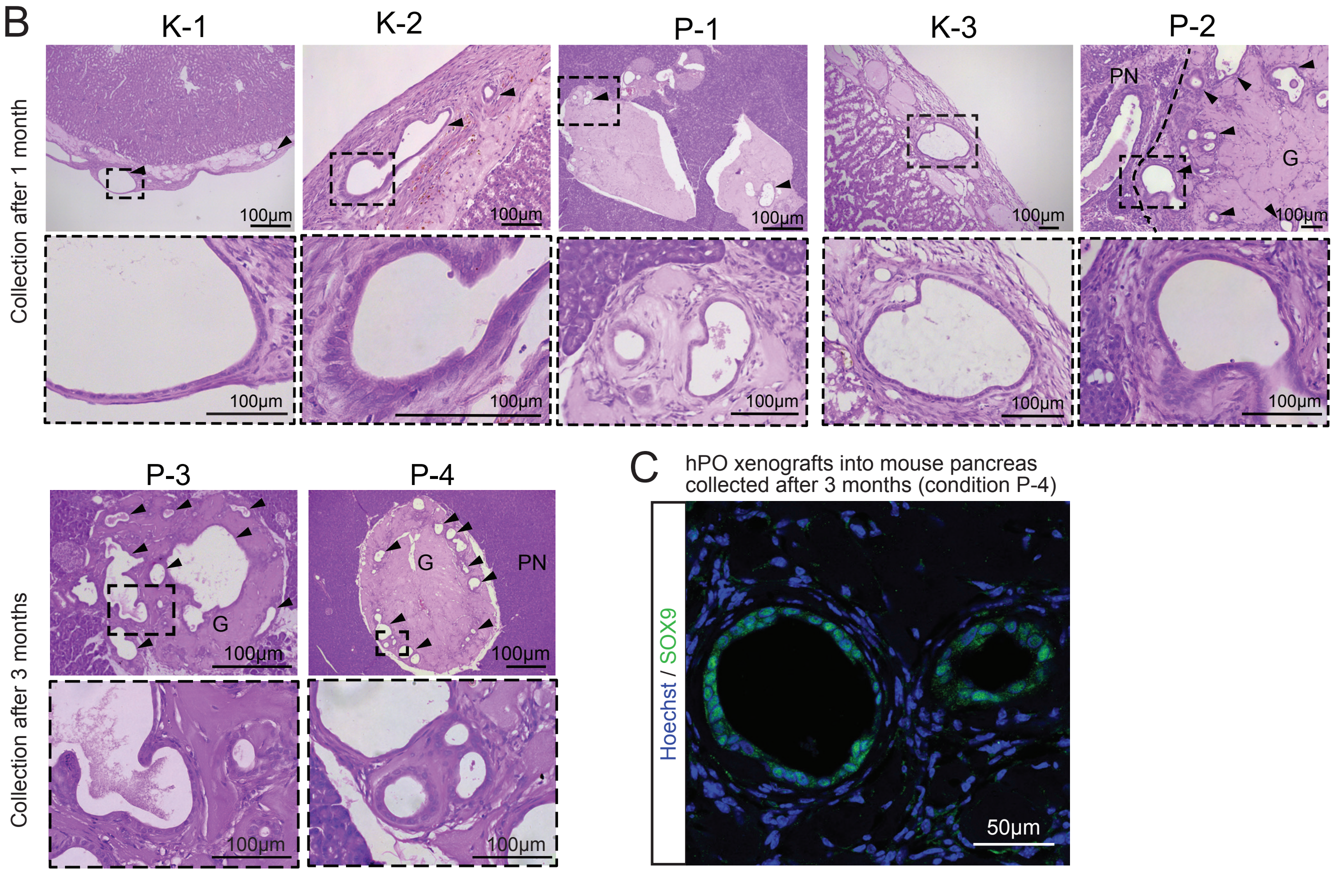

Supplementary Figure 5 - hPOs cultured in optimised hPO-Opt.EM medium grow in a chemically defined hydrogel.

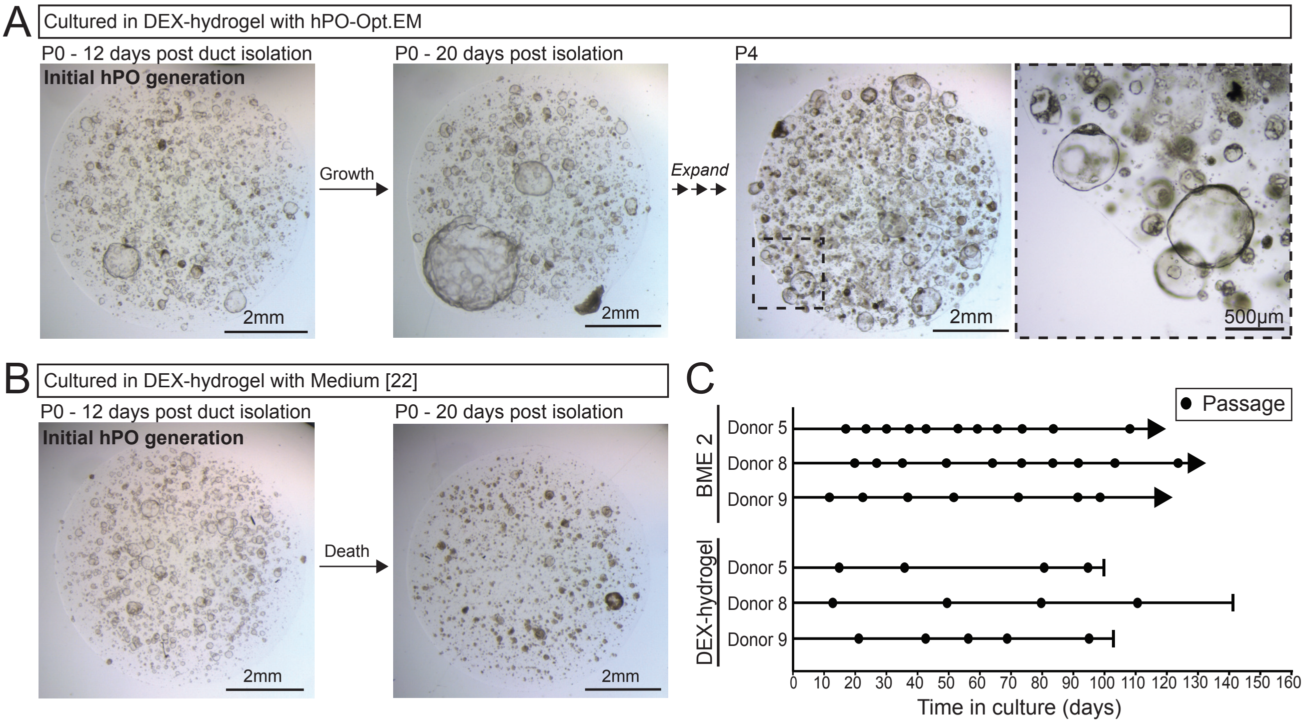

Supplement: Supplementary file 1 — Additional file 1: Figure S1. Optimisation of hPO-Opt.EM culture medium and its expansion potential compared with published pancreatic organoid culture systems. A) To obtain an optimised medium to support hPO isolation and growth, incremental changes were made to previously published protocols. Expansion graphs show the time hPOs survived in vitro for n = 4 donors in mouse pancreatic organoid (mPO) medium [20] supplemented with TGFb inhibitor (top graphs) or in our previously reported medium supplemented with TGFb inhibitor, PGE2 and Wnt-conditioned medium containing 10% serum [23] (middle graphs) or in this new optimised, chemically-defined medium (hPO-Opt.EM) containing TGFb inhibitor, PGE2, FSK and no-FBS (bottom graphs). The ability of the hPOs to expand is indicated by passaging events (cirlces), arrows indicate ongoing cultures, capped lines indicate cultures that deteriorated. While the first two conditions were not able to sustain long-term culture, the hPO-Opt.EM medium demonstrates a much greater expansion potential. B-D) Comparison of the hPO-Opt.EM medium to the chemically-defined hPO medium published by Loomans and colleagues during the course of this project [22]. Although both media enable the initial generation of hPOs, the hPO-Opt.EM medium supports long-term culture to a much greater extent than Medium [22]. B-C) Representative images of hPO cultures derived from fresh pancreas tissue using the the hPO-Opt.EM medium or Medium [22] at B) passage 0 (P0, 8-days post derivation; magnification in lower panels; n = 3), or C) at Passage 3 (top) and Passage 4 (bottom) in two independent donors. D) Graph shows the expansion potential of hPOs cultured with the hPO-Opt.EM medium or the medium published by Loomans and colleagues [22] (arrows indicate ongoing cultures, capped lines indicate cultures that deteriorated). Figure S2. Human pancreas organoids (hPOs) can be derived from fresh and cryopreserved pancreas tissue and are amenable for genetic manipulation [file 12861_2020_209_MOESM1_ESM.pdf]
